# coupled Hydrodynamic Flow Focusing (cHFF) to Engineer Lipid–Polymer Nanoparticles (LiPoNs) for Multimodal Imaging and Theranostic Applications

**DOI:** 10.3390/biomedicines10020438

**Published:** 2022-02-14

**Authors:** Felicia Roffo, Alfonso Maria Ponsiglione, Paolo Antonio Netti, Enza Torino

**Affiliations:** 1 Department of Chemical, Materials and Production Engineering (DICMaPI), University of Naples Federico II, P.le Tecchio 80, 80125 Naples, Italy; felicia.roffo@unina.it (F.R.); alfonsomaria.ponsiglione@unina.it (A.M.P.); nettipa@unina.it (P.A.N.); 2 Interdisciplinary Research Center on Biomaterials, CRIB, University of Naples Federico II, P.le Tecchio 80, 80125 Naples, Italy; 3 Center for Advanced Biomaterials for Health Care, CABHC, Istituto Italiano di Tecnologia, IIT@CABHC, Largo Barsanti e Matteucci 53, 80125 Naples, Italy

**Keywords:** microfluidics, hydrodynamic flow focusing, hybrid nanoparticle, lipid–polymer, liposome, drug delivery, theranostics, nano-bio interactions

## Abstract

An optimal design of nanocarriers is required to overcome the gap between synthetic and biological identity, improving the clinical translation of nanomedicine. A new generation of hybrid vehicles based on lipid–polymer coupling, obtained by Microfluidics, is proposed and validated for theranostics and multimodal imaging applications. A coupled Hydrodynamic Flow Focusing (cHFF) is exploited to control the time scales of solvent exchange and the coupling of the polymer nanoprecipitation with the lipid self-assembly simultaneously, guiding the formation of Lipid–Polymer NPs (LiPoNs). This hybrid lipid–polymeric tool is made up of core–shell structure, where a polymeric chitosan core is enveloped in a lipid bilayer, capable of co-encapsulating simultaneously Gd-DTPA and Irinotecan/Atto 633 compounds. As a result, a monodisperse population of hybrid NPs with an average size of 77 nm, with preserved structural integrity in different environmental conditions and high biocompatibility, can be used for MRI and Optical applications. Furthermore, preliminary results show the enhanced delivery and therapeutic efficacy of Irinotecan-loaded hybrid formulation against U87 MG cancers cells.

## 1. Introduction

A wide range of continuous microfluidic processes has been implemented for functional material synthesis [1,2] to take advantage of microfluidic peculiarity. In particular, Hydrodynamic Flow Focusing (HFF), in the forms of emulsion and solvent extraction, has been widely exploited to produce micro- and nanoparticles [3]. Furthermore, several microfluidic X-shaped geometries were investigated to synthesize polymer nanocarriers mainly consisting of chitosan [4], hyaluronic acid [5,6] and poly(lactic-co-glycolic acid) PLGA [3,7].

Firstly, Jahn and co-workers [8] synthesised liposomes in a microfluidic Y-shaped device where a lipid stream is hydrodynamically sheathed between two oblique buffer streams. Afterward, the microfluidic ability to promote the ordered interaction among lipid and polymer materials was exploited to produce hybrid architectures [9], which depending on component configurations, are classified as lipid–polymer or polymer–lipid architectures [10]. In particular, the lipid–polymer architectures combine the biodegradable core of polymer with the lipid-layer or bilayer, analogues of biological membranes, enhancing the encapsulation efficacy and offering superior biocompatibility [11] and cell uptake (nano-bio interactions) [12]. Hong et al. [13] combine the on-chip formation of liposomes encapsulating polymer precursors with off-chip polymerisation to form a hydrogel core within the liposomes. Valencia et al. [14] investigated the conditions under which stable lipid–PLGA nanoparticles were obtained in a tesla micromixer. Moreover, they implemented the same technology to synthesize NPs for imaging applications by entrapping quantum dots in lipid–PLGA NPs. Indeed, the microfluidic features and their operation conditions were exploited to enhance the controllability and homogeneity of active agent distribution with the NPs [9]. Kim and colleagues [15], developed high-density lipoprotein-mimicking NPs in microfluidics for theranostic applications, which incorporate the Simvastatin drug, a fluorescent hydrophobic agent, quantum dots, gold and iron oxide NPs. Later, Mieszawska et al. [16] have used a microvortex microfluidic platform to produce theranostic hybrid polymer−lipid NPs that load diagnostic nanocrystals and cytotoxic drug doxorubicin (DOX) in the PLGA core, while an anti-angiogenic drug Sorafenib (SRF) is placed in the lipidic layer.

These aforementioned hybrid complex lipid–polymer nanoparticles, out of all other architectures, represent a next-generation of vehicles potentially capable of overcoming the technological limitations of polymer and liposomes as standalone carriers [17]. 

The multiple step-methods for their preparation are associated with poor control and low-yield over the final carrier physical properties, relying on the addition of pre-formed polymer nanoparticles during the liposome formation or to pre-formed liposomes, requiring post-processing steps to improve the size and homogeneity of the formulation [17,18,19]. 

The inability to achieve reproducible and on-demanding properties of NPs strongly limits their performance in clinical translation [20]. Indeed, to date, the main hurdles related to the clinical development of NPs are correlated to their optimal design to overcome biological barriers and the large-scale traditional manufacturing of complex synthesis procedures [12,21]. On the contrary, the miniaturisation [22] and eventually the parallelisation [23] of batch systems down to a few centimetre squares through a microfluidic device lead to a homogeneous reaction environment obtaining a fine-tuning of the process parameters and, therefore, the control of NPs properties in a highly reproducible manner [24].

In this framework, we propose a microfluidic platform as a one-step strategy to engineer biocompatible Lipid–Polymer NPs (LiPoNs) by coupled Hydrodynamic Flow Focusing (cHFF), which allows manipulating the mutual diffusion of species and managing both nanoprecipitation and self-assembly, guiding the lipid–polymer coupling. We present a hybrid lipid–polymeric tool with a core–shell structure, where a bilayer shell made of phosphatidylcholine and cholesterol is electrostatically anchored to a polymeric chitosan core. The biomimetic behaviour of lipid material allows for optimising the surface and delivery properties of the chitosan matrix, thus improving the ability to interface and interact with cell membranes. The chitosan provides a high mechanical and colloidal stability, protects and tunes the release properties of the entrapped compounds, assists and improves their transport across the cell. Furthermore, the cholesterol addition to our liposomal formulation aims to control the fluidity of the bilayer structure, resulting in improved biostability, tumour/cell penetration [25,26] and permeability inside the body [10]. 

We exploit the multifunctional architecture of LiPoNs to co-entrap, in a single vector, multiple active agents to be applied in Multimodal Imaging (Gd-DTPA and Atto 633) and Theranostics (Gd-DTPA and Irinotecan). 

Among different diagnostic techniques, we addressed Gadolinium-based Contrast agents (CAs) for Magnetic Resonance Imaging (MRI) because, even though MRI represents the most clinically applied modality [27], Gadolinium-free CAs still suffer from transmetalation, nephrotoxic effects and abnormal brain deposition [28,29,30]. Moreover, the simultaneous combination of MRI CAs with an Optical Imaging (OI) probe improves the sensitivity of the MRI imaging techniques and potentially allows a more accurate diagnosis [31].

Furthermore, the same LiPoNs tool is validated on U87 MG cells, as a theranostic vector, by co-loading Irinotecan, as a model drug, and Gd-DTPA, for a diagnostic and therapeutic purpose. Irinotecan hydrochloride (IRI) was selected because it is already approved for a broad spectrum of human tumours [32] and is currently under repositioning for the Glioblastoma Multiforme (GBM) treatment [33,34]. 

## 2. Materials and Methods

### 2.1. Materials

L-α-Phosphatidylcholine from soybean ≥99% (SPC; lyophilised powder; storage temperature −20 °C; approximately Mw = 776 g/mol) and Cholesterol ≥99% (Chol; powder; storage temperature −20 °C; Empirical Formula C27H46O; Mw = 386.65 g/mol) have been purchased from Sigma-Aldrich (St. Louis, MO, USA). Chitosan (CH; powder; Low Mw = 50,000–190,000 Da; soluble in dilute aqueous acid); Diethylenetriaminepentaacetic acid gadolinium (III) dihydrogen salt hydrate (Gd-DTPA; Mw = 547.57 g/mol), Atto 633 (λex/em = 633/657 nm, Mw = 652 g/mol) and Atto 488 (λex/em = 480–515 nm, Mw = 804 g/mol) have been purchased from Sigma-Aldrich (St. Louis, MO, USA). Irinotecan HCl Trihydrate (IRI, Mw = 667.18 mg/mL, λabs = 368 nm) was purchased by Selleckchem Chemicals (Huston, TX, USA). As solvents, we used Acetic acid glacial (AcOH, ≥99.8%; Empirical formula CH_3_COOH; Mw = 60.052 g/mol, ROMIL pure chemistry, Cambridge, UK), Ethanol (etOH, puriss. p.a., absolute, ≥99.8%GC; Empirical formula C_2_H_5_OH; MW: 46.07 g/mol; Carlo Erba Reagents, Italy) and filtered MilliQ water (Milli-Q Plus, Q-POD^®^, Merck KGaA, Darmstadt, Germany) for all the experiments. The phosphate-buffered saline (PBS, tablet) for dialysis, cell-culture and in vitro studies was purchased by Sigma-Aldrich (St. Louis, MO, USA). CellMask Orange Plasma membrane Stain (λex/em = 554/567 nm) was purchased from Thermofisher Scientific (Altrincham, UK). The human glioblastoma cell line U87 MG (passage 30–40) was purchased from ATCC (Manassas, VA, USA). For cell culture and in vitro studies, we have used Dulbecco Modified Eagle medium-high glucose (DMEM), foetal bovine serum (FBS), Dimethyl sulfoxide for molecular biology (DMSO), Thiazolyl Blue Tetrazolium Bromide soybean ≥97.5% (MTT) and Trypan Blue purchased from Sigma-Aldrich Co. (St. Louis, MO, USA), Antibiotic Solution 100× liquid purchased and L-glutamine (200 mM) from Himedia (Einhausen, Germany).

### 2.2. Microfluidic Set-Up for coupled Flow Focusing Approach

A quartz microfluidic device (22.5 mm long × 15 mm wide × 4 mm thick) with 5 parallel inputs and one output purchased from Dolomite Centre Ltd. (Royston, UK) was used to perform all the experiments. The device consists of 5 parallel inlets converging and intersecting the corresponding end of the central channel at an angle of 45° at the junction, followed by a straight output channel. All channels have the same approximately circular cross-section of 160 μm × 150 μm. Only three of five inlets of the device are used for LiPoNs production. The chip is compatible with the H interface 7-way (Dolomite microfluidics, Royston, UK) for tubing connections. The device is connected to a glass middle syringe of 2.5 mL and glass side syringes of 5/10 mL (CETONI GmbH, Korbussen, Germany) with two PTFE tubing segments (Outside diameter OD × Inside diameter ID-1/16 mm × 0.25 mm–0.8 mm× 0.25 mm) controlled by a low-pressure syringe pump (Low-Pressure Syringe Pump neMESYS 290 N, CETONI GmbH, Korbussen, Germany). Two-way in-line ETFE valves, connecting syringes with the microfluidic device, make the automatic fill-in of the syringes feasible, thus allowing a continuous dispensing of reagents. A PTFE outlet tube (OD × ID-0.8 mm × 0.25 mm), which starts from the output of the device, was employed to collect fluid in a glass vial containing water. The flow focusing behaviour on the microchannel was observed using an Optical Fluorescence Microscope (Olympus IX71) with a 4× scanning objective.

### 2.3. One Step HFF for Lipid Polymer Nanoparticles (LiPoNs) Production

A microfluidic process was used to produce a complex nanostructure named LiPoNs. The first step consisted of preparing the etOH/Water solution (65/35% *v*/*v*) containing 0.0072% *w*/*v* of Lipids (mass ratio 8:1-SPC:Chol). It was kept under continuous stirring overnight and then injected through the side channels. The water phase is made of an aqueous solution of 0.01% *w*/*v* of CH and 1% *v*/*v* of AcOH. It was kept under continuous stirring for at least 1 h and then injected through the middle channel. To prepare Gd-DTPA-loaded LiPoNs, the contrast agent at a concentration of 0.4% *w*/*v* was added to the acid solution containing chitosan (0.01% *w*/*v*). Atto 633, Atto 488 and Irinotecan co-encapsulation was achieved by dissolving the active agents in the acetic acid solution containing AcOH-CH-Gd-DTA (1% *v*/*v*–0.01% *w*/*v*–0.4% *w*/*v*). The Atto 633, Atto 488 and Irinotecan concentrations were 24 μg/mL, 32.2 μg/mL and 145 μg/mL, respectively. Different flow rates have been tested, and the optimal Flow Rate Ratio FR^2^ (0.073), defined as the ratio of the Volume Flow Rate of the middle channel (3 μL/min) and the Volume Flow Rate of the side channel (41 μL/min), was determined for all formulations. The microfluidic process was carried out 40 min or its multiples, and the nanoparticles were collected in a vial glass containing 3.5 mL of water or its multiples. The suspension was stirred for 40 min at room temperature. Each experiment has been repeated at least ten times. For further operative conditions see Supplementary Information. 

### 2.4. Purification and Concentration of NPs

Purification was performed by solvent gradient dialysis in PBS diluted in water (1:2). The suspension was loaded in Spectra-Por Float-A-Lyzer G2, red (Molecular Weight Cut Off, MWCO = 20,000–50,000 Da, Sigma-Aldrich, St. Louis, MO, USA) or Spectra/Por™ 6 Dialysis Membrane Pre-wetted RC Tubing (Molecular Weight Cut Off, MWCO = 25,000–50,000 Da, Thermofisher, Altrincham, UK), and it was kept under continuous stirring at 230 rpm at room temperature for at least one hour. LiPoNs were concentrated by Rotary Evaporator (BUCHI Italia s.r.l, Cornaredo, Italy) at a vacuum pressure of 20 mbar at 25 °C until the desired concentration was achieved. In the case of the cell viability assay where a high concentration of material is required (Lipids concentration ≃ 1 mg/mL), additional dialysis by rotary evaporator, at the above-mentioned conditions, was performed to remove any residual solvent.

### 2.5. Characterisation of LiPoNs Nanoparticles

#### 2.5.1. Physicochemical Characterisation

Dynamic light scattering (DLS) was used to determine nanoparticle size (Zeta sizer, Malvern Panalytical, Malvern, UK). The wavelength of the laser is 633 nm, and the scattering angle used is 173°. The volume of the sample suitable for DLS analysis is 1 mL in a polystyrene cuvette (Optical Cuvette, Sarstedt, Italy). In DLS analysis, the z-Average value and the polydispersity index of the average of three measurements were collected. The DLS analyses were performed at 25 °C or 37 °C according to the aim of the analysis. Zeta potential measurements were also performed at a temperature of 25 °C on a Zetasizer Nano ZS (Malvern Panalytical, UK), loading the high-concentration surface zeta potential cell (Malvern Panalytical, UK) with 1 mL of the NP suspension.

#### 2.5.2. LiPoNs Imaging 

A Field Emission Scanning Electron Microscope (FE-SEM, Ultraplus Field Emission, Carl Zeiss, Oberkochen, Germany) and Transmission Electron Microscope (TEM, FEI, Hillsboro, OR, USA) in DRY, CRYO modes and confocal microscope (Leica Microsystems, Wetzlar, Germany) were used to morphologically characterize the nanosystem. The SEM observations were conducted by dropping 20 μL of the nanoparticle suspension on circular coverslips (22 mm), which were then air-dried overnight. Nanoparticles are coated with 5 nm Au prior to the observation. The TEM analyses were conducted both in DRY and CRYO modes. In the DRY mode, the samples were prepared using Formvar/Carbon 200-mesh Cu Agar Scientific Ltd. (Stansted, UK) using 8 μL of the suspended nanoparticles with and without staining. Negative staining is performed using a phosphotungstic acid solution (2% *w*/*v*) directly made on the deposit for 45 s or using osmium tetroxide smoke (1% *v*/*v*) overnight. Then, the air-dried samples were directly examined under the TEM. In CRYO mode, the samples were prepared using VITROBOT FEI (Hillsboro, OR, USA) coating Formvar/Carbon film 200-mesh Cu Agar with 3 μL of the nanoparticle suspension. The conditions of VITROBOT are blotting time of 3 s, humidity higher than 95% and a temperature of 4 °C. The nanoparticles were stained with CellMask Orange Plasma membrane Stain (dilution 1:10^4^) for 10 min, and then 10 μL of that solution was dropped on 25 mm FluorDish. The stained and not-stained NPs were observed using a TCS SP5 Confocal Laser Microscope with a 60 × Oil objective. Lasers with different wavelengths were used for CellMask Orange Plasma membrane Stain, Atto 633 and Atto 488 dyes at 543 nm, 633 nm and 488 nm for the excitation and at 610 nm, 657 nm and 515 nm for the emission, respectively.

#### 2.5.3. In Vitro MRI

The relaxometric properties of blank nanoparticles (LiPoNs) and nanoparticles containing Gd-DTPA (Gd-DTPA LiPoNs, Atto 633-loaded LiPoNs (Atto633 LiPoNs), Atto 633 co-loaded Gd-DTPA LiPoNs (Atto633-Gd-DTPA LiPoNs), Irinotecan-Gd-DTPA co-loaded LiPoNs (IRI-Gd-DTPA LiPoNs)) were tested by in vitro MRI. The data were compared with Gd-DTPA calibration curves dispersed in water (Figure S9a) and in water/ethanol (70/30% *v*/*v*) ranging from 0 to 100 μM and 0 to 120 μM, respectively. A total of 300 μL of NPs and diluted NP suspension in water (1:2) were dropped in an NMR tube, and the changes in relaxation time (T_1_) were evaluated at 1.5 Tesla by a Minispec Bench Top Relaxometer (Bruker Corporation, Billerica, MA, USA) at 37 °C. The relaxation time distribution was obtained using the CONTIN Algorithm, and the relaxation spectra were normalised with respect to the CONTIN processing parameters. The integral of a peak corresponds, therefore, to the contribution of the species exhibiting this peculiar relaxation to the relaxation time spectrum. 

### 2.6. Evaluation of Co-Encapsulation Efficiency

Inductively Coupled Plasma (ICP-MS) NexION 350 measurements, PerkinElmer Inc. (Waltham, MA, USA), was used to assess the concentration of the Gd^3+^ ions loaded in the LiPoNs’ suspension. The Syngistix for ICP-MS Nano Application Software Module was used to acquire and process the data. Gadolinium has been measured at m/z 157 using a 100 μs dwell time with no settling time. The Multiplate Reader Photometer Enspire Perkin-Elmer Inc. (Waltham, MA, USA) was used to quantify the encapsulation efficiency (EE %) of Atto 633, Atto 488 and Irinotecan. The absorbance (λabs = 368 nm) of the IRI-Gd-DTPA LiPoNs was correlated to the IRI calibration curve at concentrations ranging from 0.5 to 100 μM (Figure S9c). The absorbance of blank LiPoNs was measured and the concentration of blank LiPoNs was subtracted from IRI-Gd-DTPA LiPoNs at the same concentration. The fluorescence of Atto 633 (λex/em = 630/651 nm) from 25 ng/mL to 4 μg/mL for Atto 633 (Figure S9b). Triplicates of each measurement were performed. The encapsulation efficiency of Gd-DTPA, the co-encapsulation of the fluorophores and Irinotecan (EE%) were calculated as:

(1) $$\mathrm{EE}\%=\frac{\;C_{M}}{C_{T}}\times 100$$

where $C_{M}$ is the measured concentration of suspension, and $C_{T}$ is the theoretical concentration used in the microfluidic process.

### 2.7. Cell Viability by MTT Assay

Human brain glioblastoma astrocytoma cells, U87 MG, were cultured in Dulbecco’s modified Eagle’s medium (DMEM), containing FBS (10% *v*/*v*), L-glutamine (1% *v*/*v*) and penicillin-streptomycin (1% *v*/*v*), at 37 °C in water-saturated air supplemented with 5% CO_2_. For the cytotoxicity measurements, 2 × 10^4^ U87 MG cells/well were plated in 96-well plates (Corning, Costar, Merck) for 24 h before the addition of the LiPoNs. A fresh medium, containing an increasing concentration of nanoparticles, was added to each well, and the cells were incubated for 24 h or 48 h, according to the study. For the evaluation of LipoNs’ biocompatibility, an increasing concentration of LiPoNs, Gd-DTPA LiPoNs and Atto633-Gd-DTPA LiPoNs (Lipid concentration: 2–42 μg/mL; chitosan concentration: 0.07–2.1 μg/mL; Gd-DTPA concentration: 3–105 μM; Atto 633 concentration: 0.01–0.3 μg/mL) was tested for 24 h. Conversely, for the evaluation of IRI-Gd-DTPA-loaded LiPoNs’ effect on cell viability with respect to free drugs, 7.7 μM of Irinotecan in LipoNs (Lipid concentration: 0.4 mg/mL, Gd-DTPA concentration: 1.25 mM) and free IRI were tested for 24 h and 48 h on 1.5 × 10^4^ U87 MG cells/well. To exclude any additional cytotoxic effects with respect to Irinotecan, the blank formulations in the same conditions (LiPoNs-Lipid concentration: 0.4 mg/mL) were tested. At the end of the incubation time, the media were then removed, the cells were washed with PBS and fresh medium containing MTT at a final concentration of 0.5 mg/mL was added to the cells. After 3 h of incubation, the medium was removed, and the insoluble formazan crystals synthesised by the live cells were dissolved in 200 μL of DMSO. After 30 min of incubation, the absorbance (λabs = 556 nm) of the solution was then recorded in triplicate using a Multiplate Reader Photometer Enspire from Perkin-Elmer Inc. (Waltham, MA, USA). The viability percentage of nanoparticle-treated cells were evaluated, with the cells not treated with nanoparticles or active agent considered as the control.

(2) $$\mathrm{Cell}\;\mathrm{viability}\;\%=\frac{\mathrm{AU}\;\mathrm{of}\;\mathrm{Cells}\;\mathrm{tested}\;\mathrm{with}\;\mathrm{NPs}\;}{\mathrm{AU}\;\mathrm{of}\;\mathrm{Untreated}\;\mathrm{Control}\;\mathrm{Cells}}\times 100$$

where the AU is the measured Absorbance in triplicate.

### 2.8. Flow Cytometry Analysis

Prior to the addition of NPs, 5 × 10^4^ U87 MG cells/well are seeded in 48-well plate (Corning, Costar, Merck) and incubated for 24 h. Afterwards, cells were incubated with culture medium containing an increasing concentration of Atto633-Gd-DTPA LiPoNs (Lipid concentration: 30–60–90 μg/mL, Gd-DTPA concentration: 75–150–225 μM, Atto 633 concentration: 0.2–0.4–0.6 μg/mL) for different time intervals according to the uptake study. The time intervals for the cellular uptake study with a Lipid concentration of 90 μg/mL in LiPoNs were set to 2 h, 4 h, 6 h, 8 h and 24 h, while for a comparative study of LiPoNs’ uptake at different concentrations (30–60–90 μg/mL Lipid concentrations), the time intervals were 4 h, 8 h and 24 h. Negative controls were the complete medium condition without NPs. Then, the medium was removed, and the samples were washed three times with PBS (1×) to ensure particle removal from the outer cell membrane. Cells were then trypsinised for 5 min at 37 °C and transferred from the cell culture medium (without phenol red) to polystyrene round-bottomed tubes (Falcon round-bottom, Thermofisher, Altrincham, UK) on ice. The samples were analysed by flow cytometry using a BD FACSCelesta Cell Analyzer (BD Biosciences, Franklin Lakes, NJ, USA). A total of 10,000 events were recorded for each sample in triplicate, and the cells were gated using Forward Scattering Area (FSC) and Side Scattering Area (SSC). For the FI distribution, the cells were excited at 561 nm, and the fluorescence emitted by Atto 633 (PE_Cy5_A, Filter 688/33) was collected in the PE_CY5_A channel. The autofluorescence of the cell line was determined by untreated cells, which were used as a control. The data obtained by BD FACSCelesta Cell Analyzer were analysed with CytoFlow software (v1.1.1, Massachusetts Institute of Technology 2015–2018, Cambridge, MA, USA). The results are reported in Figures S8–S10 as the geometric mean of Fluorescence Intensity (FI), Side Scattering (SSC) and Forward Scattering (FSC), and the error bars are the standard deviations between the replicates. The raw flow cytometry data of U87 MG cells positive (orange) or not (blue) to a threshold gate for PE_ Cy5_A channel are reported in Figures S13–S15.

### 2.9. Cell Uptake Study by Confocal Imaging

The Atto633-Gd-DTPA LiPoNs cellular uptake was studied by confocal imaging. First, 5 × 10^4^ U87 MG cells/well were seeded in µ-Slide 8-Well (Ibidi, Bayern, Germany) and cultured for 24 h. The medium was removed, and a fresh medium with Atto633-Gd-DTPA LiPoNs (Lipids: 90 μg/mL, Gd-DTPA: 225 μM, Atto 633: 0.6 μg/mL) was added and incubated for 24 h. For the cell membrane staining, CellMask Orange Plasma membrane Stain (dilution 1:10^5^) was added to the cells for ≃15 min and then replaced with PBS for live acquisition. Live cells were observed with a Leica Microsystems TCS SP5 Laser Scanning Confocal Microscope (Wetzlar, Germany) with a 60 × Oil objective. Atto633-Gd-DTPA-loaded LiPoNs were excited with the HeNe 633 nm laser, and bight field was used to assess cell morphology. The Z-Stack videos were recorded by acquiring one image every 15 s for up to ≃8 min for cells exposed to Atto633-Gd-DTPA co-loaded LiPoNs (Supplementary Videos S2 and S3).

## 3. Results

### 3.1. coupled Hydrodynamic Flow Focusing (cHFF) for LiPoNs Production

A well-established technique for liposome production is thin-film hydration, which consists of swelling dry phospholipid films in excess water under vigorous shaking, inducing spontaneous vesicle self-assembly [35]. However, this spontaneous bulk process is unable to control the physical properties of the end-products [24]. Recently, microfluidic systems, outdoing the above-mentioned limitation, offer control over the confinement microenvironment with a dimension of the nanoparticle itself [8]. In these systems, specific nanostructures are formed by the Hydrodynamic Flow Focusing (HFF), where the non-solvent phase, flowing through two side channels, focuses the solvent phase in the middle channel [2].

**Figure 1:**
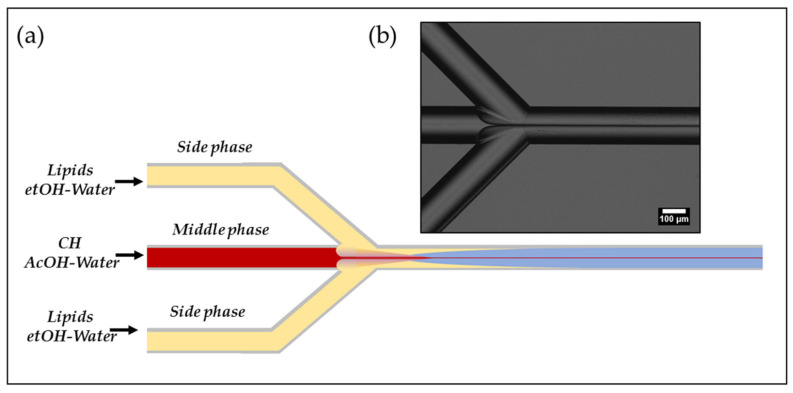
Schematic illustration of microfluidic experimental set-up: (**a**) Qualitative illustration of coupled Hydrodynamic Flow Focusing (cHFF) strategy processed in the microfluidic device; (**b**) Transmission Optical Microscopy Image of Hydrodynamic Flow Focusing (HFF) pattern.

**Figure 2:**
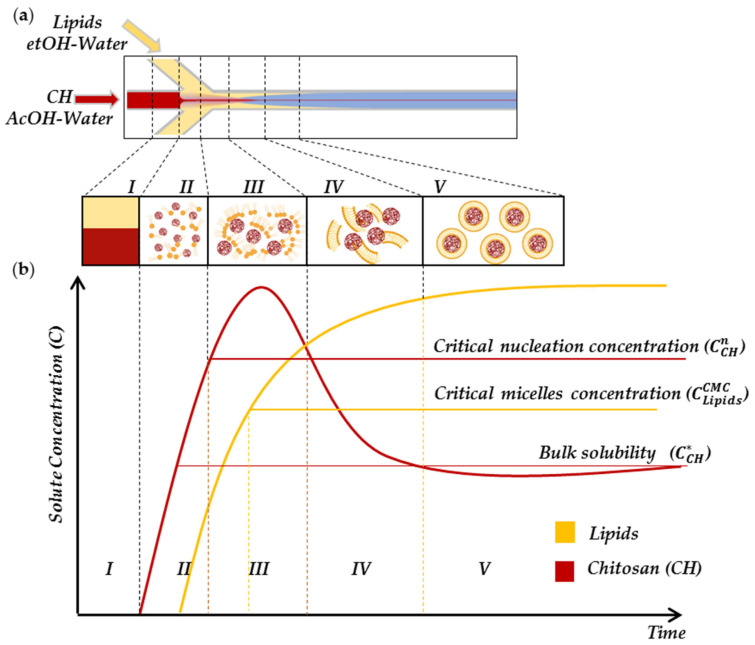
Schematic illustration of nanoprecipitation and self-assembly processes implemented in microfluidics. (**a**) Sketch of coupled Hydrodynamic Flow Focusing (cHFF), implemented by injecting Lipids dissolved in Ethanol/Water (etOH/Water, yellow stream) and Chitosan in Acetic Acid/Water (AcOH/Water, red stream) for Lipid–Polymer Nanoparticles’ (LiPoNs) production (blue stream). (**b**) Schematic diagram of LiPoNs forming process in the cHFF: (I) Chitosan and Lipids dissolved in solvent mixture injected into the microfluidic channels; (II) Chitosan supersaturation and Lipid monomers formation; (III) Chitosan Nuclei and Lipid micellization; (IV) Chitosan intermediate and bilayer fragments formation; (V) Lipid–chitosan nanocomplex production.

Here, coupled Hydrodynamic Flow Focusing (cHFF), as schematised in Figure 1, is proposed for the first time to produce a complex lipid–polymer nanosystem named LiPoNs. The ability to manage two thermodynamic processes is obtained simultaneously by controlling the time scales of solvent exchange [24,36], to induce the polymer precipitation and the self-assembly of bilayer fragments that coat the NPs’ surface The peculiarity of the proposed approach for the synthesis of LiPoNs (Figure 1 and Figure 2a,b, region I) relies on the injection of two lateral lipid streams, dissolved in a variable ethanol–water (etOH/Water) ratio, that squeeze the chitosan (CH), dissolved in acetic acid (AcOH) solution, injected into the middle channel. On the contrary, in the literature, the formation of liposomes is typically performed by injecting the lipid solution into the main channel [8,37,38].

The cHFF features govern the competition of two solvent extractions and therefore coordinate the relative kinetics of nuclei and the growth of two phenomena: nanoprecipitation and self-assembly. The steps of cHFF involve a rapid nucleation rate of chitosan, self-assembly of lipids in bilayer fragments and, finally, the coupling of chitosan with the bent bilayer fragments. Consequently, the rapid chitosan precipitation mediates the bilayer fragments’ enclosure. Indeed, the designed cHFF leverages rapid acetic acid (AcOH) extraction that promotes fast nucleation, leading to almost monodisperse chitosan nanoprecipitate (Figure 2a,b, region II). Simultaneously, once the lateral solution comes in contact with the middle flow, the lipids are no longer solubilised and begin to assembly in bilayer fragments due to the organic solvent extraction (Figure 2a,b, region III–IV). Then, the already formed bilayer fragments (slightly negatively charged) diffuse to the polymer nuclei nanoparticles (positively charged), covering their surface and inhibiting the further growth of chitosan nuclei, finally stabilising the LiPoNs complex. (Figure 2a,b, region V). 

### 3.2. Microfluidic Platform to Obtain coupled Hydrodynamic Flow Focusing (cHFF) and the Optimisation of Process Parameters to Control the LiPoNs’ Nanostructures 

We investigated the process parameters in terms of fine-tuning the flow rates, solvent–nonsolvent ratio, solute concentration and ${\mathrm{FR}}^{2}$, which govern the coupling time of thermodynamic phenomena: nucleation of chitosan particles, self-assembly of lipid fragments and final interaction of these intermediate structures. In this work, FR^2^ is defined as follows:

(3) $${\mathrm{FR}}^{2}=\frac{\mathrm{Volume}\;\mathrm{Flow}\;\mathrm{Rate}\;\mathrm{of}\;\mathrm{AcOH}/\mathrm{Water}\;\mathrm{solution}\;\left(\mathrm{Middle}\;\mathrm{phase}\right)}{\mathrm{Volume}\;\mathrm{Flow}\;\mathrm{Rate}\;\mathrm{of}\;\mathrm{etOH}/\mathrm{Water}\;\mathrm{solution}\left(\;\mathrm{Side}\;\mathrm{phase}\right)}$$

A preliminary study was performed (Table S1) evaluating the effect of solvent-non solvent ratio (etOH/Water: 80/20% *v*/*v* and 65/35% *v*/*v*; AcOH/Water: 1/99% *v*/*v* and 10/90% *v*/*v*) and the concentration of the reagents (Lipids: 0.016% *w*/*v* and 0.0072% *w*/*v*; chitosan: 0.01% *w*/*v* and 0.0375% *w*/*v*) for a proper interaction of both Lipids and chitosan components. Then, the effect of the FR^2^ on the morphology of LiPoNs was assessed. A feasibility study was conducted by ranging FR^2^ from 0.024 to 0.68, which was obtained by keeping the lateral flow rate at 41 µL/min and increasing the middle one from 1 to 28 µL/min. Finally, the effect of the collection volume was also analysed by considering a collection volume of 2, 3.5 and 8 mL. Detailed results of the entire experimental campaign analysed by TEM and DLS are reported in Table S1 and Figures S1–S6.

**Figure 3:**
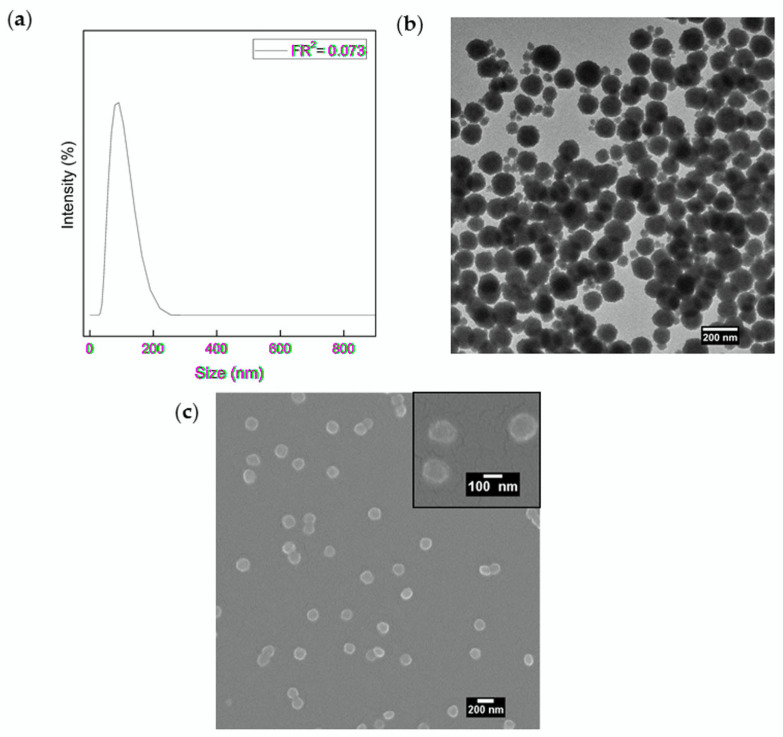
LiPoNs size distribution and morphological characterisation: (**a**) Particle Size Distribution, (**b**) TEM image (stained with osmium smoke) and (**c**) SEM image of LiPoNs performed at optimal conditions, with a Lipids concentration of 0.0072% *w*/*v* (mass ratio 8:1 SPC:Chol) dissolved in etOH/Water (65/35% *v*/*v*) and a CH concentration of 0.01% *w*/*v* dissolved in acid solution (AcOH-1% *v*/*v*) at FR^2^ of 0.073.

The stability of the microfluidic process and the absence of massive precipitation combined with the evaluation of the morphologies guided the identification of the main operative conditions to obtain stable structures. As a result of optimisation studies for LiPoNs’ synthesis, the value of FR^2^ equal to 0.073, (obtained at a middle flow rate of 3 μL/min, a side flow rate of 41 μL/min), a collection volume of 3.5 mL, a chitosan concentration of 0.01% *w*/*v* (dissolved in acetic solution 1% *v*/*v*-middle phase) and a Lipid concentration of 0.0072% *w*/*v* (dissolved in etOH/Water-65/35% *v*/*v*-side phase), were proven to be the optimal conditions and were further used for all the experiments. The results on LiPoNs obtained with these optimal conditions were compared in terms of z-Average, particle size distribution (PSD), polydispersity index (PDI) and standard deviation (St.Dev) value and zeta potential obtained by Zetasizer Nano and are reported in Figure 3a and Table 1. 

A monodisperse population of LiPoNs is observed by TEM in Figure 3b, where a nanostructured system composed of a dark core, due to the entrapment of the polymer within a less electrodense lipidic coating, is shown. Other core–shell nanostructures observed by cryo-TEM are reported in (Figure S7a). The size and homogeneity of LiPoNs by SEM image are reported in Figure 3c and Table 1. To confirm the presence of the lipid-shell in the LiPoNs’ structure, confocal images are also performed to support the morphological characterisation provided by TEM images. The confocal microscope observations of LiPoNs, stained with CellMask Orange Plasma, were performed. Red fluorescent spots are detected in Figure S7b.

These results confirm the importance, as reported in recent studies [39,40,41], of considering the effect of a whole microfluidic channel path on NPs morphologies. Indeed, all the operative conditions, from the pre- and post-injection to the mainstream behaviour and collection volume contribute to the thermodynamic formation and stability of the nanoparticles. 

### 3.3. Stability Study of LiPoNs Nanoparticles

**Figure 4:**
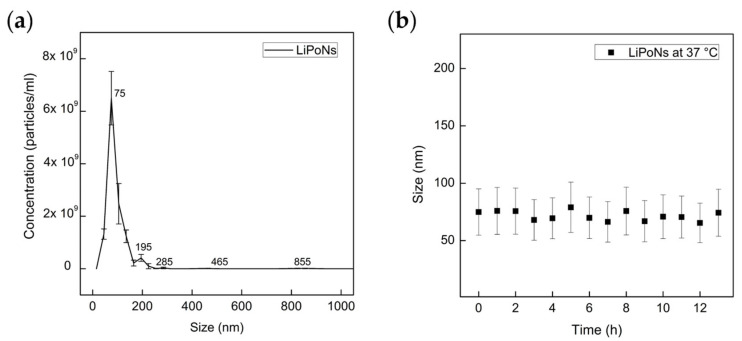
Physical Stability Study of LiPoNs: (**a**) particle size distribution is expressed as the average and standard error of the mean LiPoNs’ concentration (particles/mL) evaluated in PBS (diluted 1:200) for five measurements; (**b**) LiPoNs’ average size and standard deviation observed at 37 °C for several time points (up to 13 h).

NTA analysis allows a dynamic observation, counting and sizing of LiPoNs and a high-resolution of their distribution [42]. The NTA results of LiPoNs obtained at optimal conditions and dispersed in PBS are shown in Figure 4a. The mean and the mode of nanoparticle size are 94.7 nm and 79.7 nm, respectively, with 90% of the nanoparticles being <137.2 ± 6.1 nm (Figure S8a–c). The real-time visualisation (Figure S8a) of LiPoNs as individual particles confirmed their stability in PBS. The nanoparticle concentration is around 1.24 × e^10^ particles/mL. Results clearly show that LiPoNs are monodisperse NPs and stable upon aggregation phenomena as observed by the NTA Video (Video S1). 

In 2018, the FDA issued a guideline for liposome drug products addressed to industry, where the need to perform stress stability tests to assess liposome size distribution and integrity is underlined [43,44]. Indeed, stress testing of unloaded liposomes is conducted to evaluate possible degradation due to hydrolysis of saturated and unsaturated lipids. It was reported [45], for formulations similar to LiPoNs, a rapid decrease in NP concentration in plasma within 11 h post-injection (with a $T_{1/2}\alpha$ of 28.08 min and $T_{1/2}\beta$ of 297.05 min). As a comparison, the LiPoNs size distribution was evaluated by DLS over time (up to 13 h) at 37 °C, and no significant increase in their average size and St.Dev was observed (Figure 4b). We hypothesize that the electrostatic interaction between the chitosan entrapment and lipid bilayer reduces the phosphate group’s motional freedom, increasing the stability [46]. 

### 3.4. Co-Encapsulation Efficacy of Multifunctional LiPoNs

The designed microfluidic process and the LiPoNs core–shell structure make this set-up suitable for active compound loading. Indeed, the payload agents are dissolved in chitosan solution and then injected into the middle channel at optimal conditions, forcing their loading in the core of the LiPoNs’ complex. Firstly, the diagnostic properties of Gd-DTPA-loaded LiPoNs (Gd-DTPA LiPoNs) were studied by adding the contrast agent to the central polymer solution (mass ratio 1:40 of CH:Gd-DTPA) of the microfluidic platform. Higher precipitation along the focused stream without instabilities at the flow-focusing interface was observed. 

**Figure 5:**
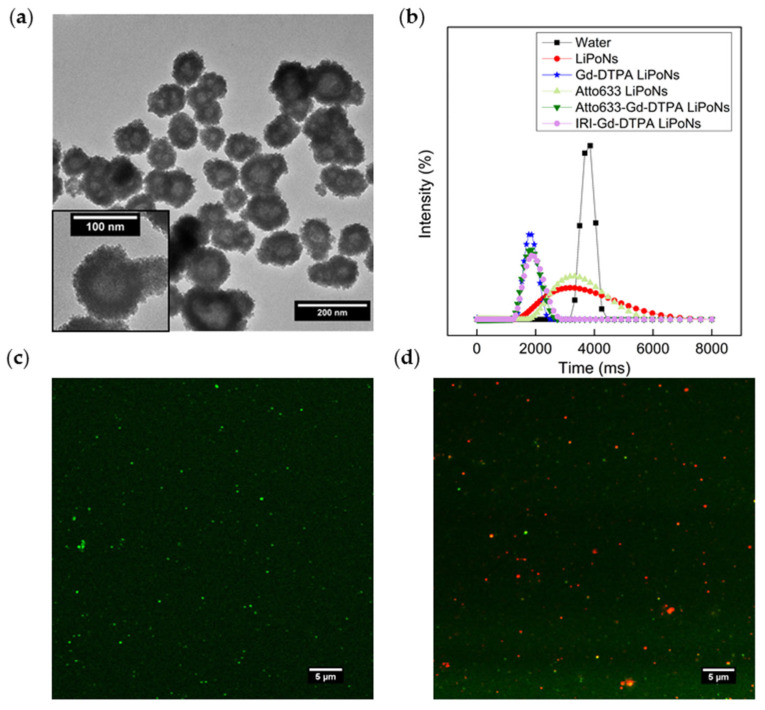
Multimodal imaging properties of LiPoNs: (**a**) TEM image of Gd-DTPA-loaded LiPoNs stained with osmium smoke; (**b**) in vitro MRI. Comparison of longitudinal relaxation time distributions of water, LiPoNs, Gd-DTPA-loaded LiPoNs, Atto633-loaded LiPoNs, Atto633-Gd-DTPA co-loaded LiPoNs, IRI-Gd-DTPA co-loaded LiPoNs, (**c**) optical imaging of Atto 488-loaded LiPoNs by confocal microscopy; (**d**) merge fluorescent image of Atto 488 (green) and CellMask (red) of Atto 488-Gd-DTPA co-loaded LiPoNs stained with CellMask™ Orange Plasma membrane stain (dilution 1:10^4^).

A slight increase in the size of LiPoNs with the loading of Gd-DTPA is outlined (Table 1). The TEM image in Figure 5a shows a less stained core of nanoparticles resulting from Contrast Agent (CA) loading within the chitosan matrix. The in vitro longitudinal relaxation time T_1_ of Gd-DTPA LiPoNs is evaluated at 37 °C and 1.5 T. The amount of Gd-DTPA entrapped in the NPs was quantified from a calibration curve with the Minispec Bench Top Relaxometer (Bruker Corporation). The data were confirmed by ICP-Ms analysis. The Gd-DTPA LiPoNs show an encapsulation efficacy of Gd-DTPA of 78% (Figure S9a and Table 1). LiPoNs show a slightly negative surface charge of −17.4 mV, which may be attributed to the replacement of a phospholipid by cholesterol (mass ratio 8:1 of SPC:Chol) [47], while Gd-DTPA LiPoNs display a zeta potential of −11 mV linked to the encapsulation of Gd-DTPA (Table 1). 

We next examined the optical and theranostic properties of LiPoNs by simultaneous encapsulation of Gd-DTPA and active agents (Atto 633/Irinotecan) alternatively. In vitro longitudinal relaxation time T1 of LiPoNs, Gd-DTPA LiPoNs, Atto 633-loaded LiPoNs (Atto633 LiPoNs), Atto 633 and Gd-DTPA co-loaded LiPoNs (Atto633-Gd-DTPA LiPoNs), Irinotecan and Gd-DTPA co-loaded LiPoNs (IRI-Gd-DTPA LiPoNs) and pure water as control is shown in Figure 5b. In the co-loading of Atto 633, a decrease in the EE% of Gd-DTPA from 78% to around 67% is observed; conversely, the co-encapsulation of Irinotecan does not influence the loading of Gd-DTPA in the IRI-Gd-DTPA LiPoNs, keeping the EE-equal to 79% (Table 1).

The amount of co-loaded Atto 633 and Irinotecan has been quantified through measurements with the Multiplate Reader Photometer starting from a calibration curve (Figure S9b,c). The co-EE% of Irinotecan is 64%, while the co-EE % of Atto 633 is 55%. No effect of co-loading of Gd-DTPA is reported on the Atto 633 loading efficacy (55%) with respect to Atto 633 alone (EE-57%). DLS measurements of Gd-DTPA LiPoNs and IRI-Gd-DTPA LiPoNs show sizes of 95.3 ± 26.35 nm and 112.8 ± 30 nm, respectively (Table 1). No impact of the entrapment of active agents on the surface charge of LiPoNs is reported, except for the loading of Atto 633, a cationic dye, which reduces the LiPoNs’ surface charge (−3.7 mV).

The optical properties of LiPoNs (Atto 488) were confirmed by the confocal observations, as shown in Figure 5c. 

To gain more insight into the structural properties of LiPoNs, we compare the loading of the fluorescent agent within the chitosan core and the CellMask Orange Plasma membrane staining of the lipid components using a confocal microscope. The overlapping of the CellMask Orange Plasma membrane stain and fluorescent spherical spots of Atto 488 and Atto 633, are shown in Figure 5d and Figure S10, respectively. 

### 3.5. In Vitro Cytotoxicity Study

**Figure 6:**
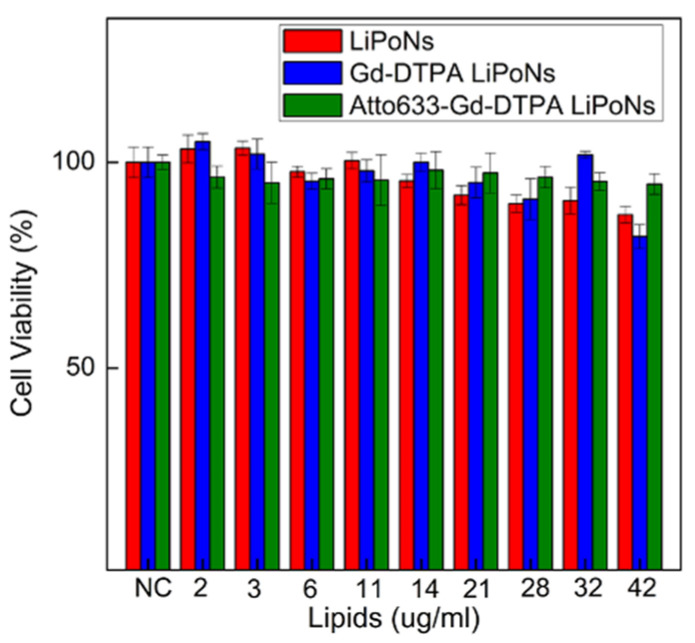
In vitro citotoxicity. Cell viability % of U87 MG cells exposed to an increasing concentration of LiPoNs, Gd-DTPA-loaded LiPoNs and Atto 633-Gd-DTPA co-loaded LiPoNs (Lipid conc.: 2–42 μg/mL, chitosan conc.: 0.07–2.1 μg/mL, Gd-DTPA conc.: 3–105 μM, Atto 633 conc.: 0.01–0.3 μg/mL) for 24 h.

Typically, lipid nanostructures are considered pharmacologically inactive compounds, even though their toxicity is related to several factors, such as the lipid composition, the surface charge and the time and the dose of exposure [44]. Indeed, different liposomal formulations [48,49] induces toxicity for 50% of exposed cells over certain concentration defined as a lethal concentration ($LC_{50\%}$) [50]. Parnham et al. [51] suggested different methods for screening the toxicity of liposomes, including the MTT assay [52]. As a reference, good biocompatibility of liposomes composed of Soy Lecithin and cholesterol with concentrations ranging from 0 to 500 μg/mL has already been reported [53]. Chitosan is a natural and biocompatible polymer, and it exhibits cytotoxicity only at concentrations higher than 0.741 mg/mL [54]. In accordance, the U87 MG cells were treated for 24 h with increasing concentrations of LiPoNs, Gd-DTPA LiPoNs, Atto 633-Gd-DTPA LiPoNs (Lipid concentration 2–42 μg/mL; chitosan concentration 0.07–2.1 μg/mL) showed no detectable cytotoxicity in vitro. The data report no significant reduction in cell viability % for all tested formulations (Figure 6). 

### 3.6. Evaluation of Cellular Uptake of Multimodal Imaging LiPoNs

**Figure 7:**
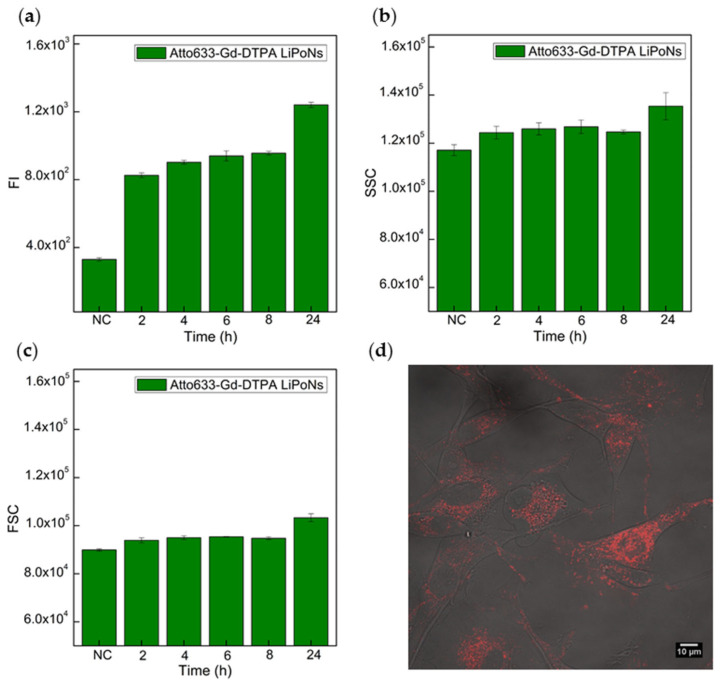
Quantitative uptake of multimodal imaging LiPoNs by U87 MG cells: (**a**) Fluorescent Intensity (FI); (**b**) Side Scattering Area (SSC); (**c**) Forward Scattering Area (FSC) of U87 MG cells exposed to Atto633-Gd-DTPA co-loaded LiPoNs (Lipids conc.: 90 μg/mL, Atto 633 conc.: 0.6 μg/mL, Gd-DTPA conc.: 225 μM) for different time points: 2 h, 4 h, 6 h, 8 h and 24 h. (**d**) Merge image of the transmission and fluorescence (Atto633) images obtained by confocal microscopy of U87 MG cells treated for 24 h with the 90 μg/mL of Atto633-Gd-DTPA LiPoNs.

A preliminary study of cellular uptake kinetics of LiPoNs is performed by fluorescence-activated cell sorting (FACs) and confocal microscope imaging. U87 MG cells, upon incubation with Atto633-Gd-DTPA LiPoNs, at a lipid concentration of 90 μg/mL, were interrogated individually for up to 24 h (Figure 7a–c). 

The fluorescence intensity of cells (Figure 7a) rises quickly (2 h) and pursues linearly over time (24 h). Samples at the same concentration were analysed by FACS in terms of Forward Scattering (FSC) and Side Scattering (SSC) intensity after incubation of Atto633-Gd-DTPA-loaded LiPoNs (Figure 7b,c). The SSC signal is known to be related to the inner complexity or granulometry of the cells and the internalisation of NPs inside the cells [55], and our data show an increase of SSC signal with longer times of incubation (Figure 7b). The FSC is related to cell size and the cell death process [55]. In this case, no changes in FSC signal are reported, reiterating the high biocompatibility of LiPoNs nanosystems (Figure 7c). These results match the FI signal already described (Figure 7a) and confirm the LiPoNs uptake kinetics. 

Further tests to evaluate the effect of the LiPoNs concentration, ranging from 30 to 90 μg/mL, on cell uptake are reported in Figure S11a–c. The analysis of the fluorescence signal of the cells treated with an increasing concentration of LiPoNs (30–90 μg/mL) provides additional insights regarding their uptake kinetics (Figure S11a). The results show a rise in the fluorescence signal in correlation with the increasing concentration of LiPoNs tested, but a more marked dose-dependent effect is observed over longer times. In the case of a lower LiPoNs concentration, the cell fluorescence increases within 4 h, and it remains almost unchanged until 24 h. The results are also coherent with SSC and FSC acquisitions (Figure S11b,c). 

Regarding uptake kinetics, all tested concentrations (Figure 7a–c and Figure S11a–c) show a high uptake rate in the first 4 h of incubation, so we hypothesize that LiPoNs uptake occurs mainly in the first 4 h. This linear increase in the mean fluorescence without any saturation phenomena at shorter times was previously reported as energy-dependent uptake [56]. The raw flow cytometry data of U87 MG cells exposed to increasing concentrations of Atto633-Gd-DTPA-loaded LiPoNs (Lipids conc.: 30–60–90 μg/mL) at different time points (4 h; 8 h; 24 h) are reported in Figures S13–S15.

We also establish the localisation of LiPoNs within the cells, imaging U87 MG cells treated with Atto633-Gd-DTPA LiPoNs, at a lipid concentration of 90 μg/mL, for 24 h with a confocal microscope (Figure 7d and Figure S12). The Merge Z-stack video of the transmission and fluorescence (Atto 633) of the cell treated with LiPoNs shows the internalisation of NPs within the cells (Video S2). 

We can hypothesize that the increase in the mean fluorescence intensity at 24 h for a higher LiPoNs concentration is mainly due to the large number of NPs still available in the extracellular medium; therefore, their uptake is continuous over time. This latter statement is confirmed quantitatively by confocal Z-stack images where LiPoNs were localised near to cytoplasmic membrane of the minority of the cells even after 24 h (Video S3). 

This finding, in accordance with Daphne Montizaan et al. [57] which reported that the uptake of zwitterionic and negatively charged liposomes, is typical of an energy-dependent mechanism, particularly clathrin-mediated endocytosis. However, the liposome-cell interaction can also occur through other three mechanisms (adsorption, lipid exchange and fusion) [58,59]. Therefore, the lipophilic interaction of LiPoNs with the cell membrane could induce a direct uptake by passive diffusion [60] in parallel to an energy-dependent mechanism. Indeed, Guo et al. [61] reported that the cell internalisation pathway shifts from fusion to endocytosis by altering the elasticity of particle nanolipogels, changing the extent of crosslinking of the core material encapsulated in the liposome. In our case, the un-crosslinked core in LiPoNs could confer a moderate elasticity to nanoparticles that could mediate the cellular uptake of both fusion and endocytosis pathways. 

These behaviours required further investigation because differences in the nanoparticle formulation (head group of lipids, cholesterol addition, size, protein corona absorption), cell type and nanostructure elasticity, which can be controlled by microfluidics, strongly affect the NPs uptake behaviour [58,59,62]. 

### 3.7. In Vitro Assessment of Cytotoxicity Activity of Theranostic IRI Gd-DTPA-Loaded LiPoNs 

To test the biological activity of theranostic Irinotecan Gd-DTPA co-loaded LiPoNs (IRI-Gd-DTPA LiPoNs) on human glioblastoma cells (U87 MG), a quantification of their cell cytotoxicity was performed. Irinotecan hydrochloride (IRI) is a semisynthetic of camptothecin used to inhibit topoisomerase-I (Topo I), producing DNA strand breaks and inducing cell death [33]. Indeed, in Phase I/II trials, Irinotecan has shown encouraging results for the treatment of malignant glioma alone or in combination with other cytotoxic drugs [63].

**Figure 8:**
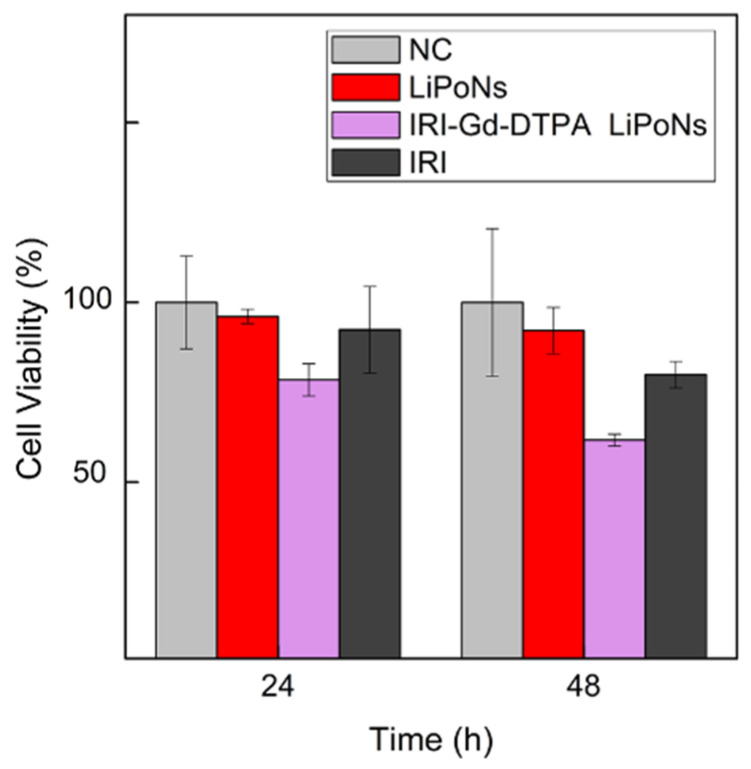
Viability of U87 MG cells treated with theranostic LiPoNs. Comparison of cell viability % of U87 MG cells treated for 24 h and 48 h with 7.7 μM of free irinotecan and in IRI-Gd-DTPA co-loaded LiPoNs formulation (Lipid conc.: 0.4 mg/mL, Gd-DTPA conc.: 1.25 mM). The cell viability % of U87 MG cells in the presence of blank LiPoNs at the same tested conditions of IRI-Gd-DTPA LiPoNs is reported.

Therefore, the MTT assay on U87 MG glioblastoma cells was used to assess the improved efficacy of the IRI-Gd-DTPA LiPoNs formulation in comparison with free drug (Figure 8) on cell survival and growth. 

U87-MG cells were treated with 7.7 μM of free Irinotecan alone and loaded in LiPoNs (IRI-Gd-DTPA LiPoNs) for different time intervals (24 h and 48 h) (Figure 8). Blank LiPoNs were tested in the same conditions as a control. 

At 24 h, in the case of IRI-Gd-DTPA-loaded LiPoNs, a reduction in cell viability down to 78.5% ± 5 was detected. Moreover, the results at 48 h show a cell survival decreased from 100 to 61.7% ± 2 and 79.9% ± 4 when the cells were incubated with IRI-Gd-DTPA LiPoNs and free IRI, respectively. The most pronounced difference in the LiPoNs’ formulation with respect to free drugs on the reduction in cell viability was observed and was highlighted at a longer incubation time of 48 h. This effect is in line with the pharmacodynamics of Irinotecan, which is a topoisomerase inhibitor, acting on cell division mainly completed up to 48 h [34].

These findings are in agreement with Casado et al. [64], who reported a different uptake mechanism for Irinotecan-loaded liposomes (mainly endocytic process) and free drugs (passive diffusion). Moreover, they highlighted the increase in the therapeutic index of Irinotecan using a liposomal formulation. 

Comparing the data shown in Figure 8 with the bibliographic reference [33], LiPoNs seem to act as efficient theranostic carriers (≃61% U87 MG cells) for a lower dose (7.7 μM) at equal exposure time (48 h). It was also reported that the entrapment of Irinotecan within a liposome core at a low pH improves the stability of the lactone, the active form of the drug, avoiding its hydrolysis at a physiological pH [64,65]. Finally, we also proved that the designed one-step cHFF enables a higher IRI encapsulation thanks to a microfluidic environment condition that locks the IRI in its active form within the architecture of LiPoNs. The acid chitosan core loads and preserves the IRI in lactone form, while the lipid counterpart mediates its cellular delivery, therefore improving its efficacy.

## 4. Discussion

Effective targeting and biological outcome of newly synthetised nanovectors are mainly determined by the dynamic and complex interplay that occurs between the heterogeneous biological microenvironment and the nanocarrier itself, defined as nano−bio interactions [66]. Even though nanoparticles are designed with an optimal size, shape and surface charge to overcome the biological barriers encountered in the delivery to a solid tumour, less than 1% will accumulate in the target site [61,67]. Recently, the impact of a new parameter, called elasticity, on the biological functions and fate of nanoparticles has been outlined [68]. It is mainly relevant for lipid-based nanoparticles where ionizable lipids enable endosomal escape and contain helper lipids to promote cell binding and cholesterol to control the fluidity of the bilayer [69]. It has been a long road to optimize the lipid-based nanostructures. Thus, microfluidic technology has the features to tackle the issue of the development of nanostructures and support the optimal design of the delivery systems, improving nanoparticle–biological interaction and guiding new designs and delivery strategies. The recent success of pharmaceutical nano-formulations is pushing towards the development of quality-oriented bottom-up manufacturing processes to face the challenging scale-up to commercial production volumes of conventional production methods [70]. Indeed, in the translation of manufacturing conditions from laboratory to an industrial scale, the conventional bulk mixing devices are characterised by variability and heterogeneity within and between batches that results in sub-optimal control of quality attributes of end-products. This discrepancy in the transition to industrial manufacturing scales could be overcome by microfluidics technology through modular and continuous production set-up allowing fine control of process parameters and their predictability even at large scale production [71,72].

In this work, we presented a new generation of lipid polymer nanoparticles obtained by coupled Hydrodynamic Flow Focusing (cHFF), modifying HFF and using it in an innovative manner to control the coupling of the lipid fragments with the polymer nuclei. The thermodynamics of the Polymer–Lipid NPs (LiPoNs) process is hypothesised in five main regions (Figure 2a,b), controlling the classic crystallisation theory for chitosan nanoparticle formation [73] and the hydrophobic effects as driving forces for liposome assembly [74].

The nanoprecipitation begins at the onset of supersaturation, generated by the sudden change in concentration, to reduce the system’s free energy (ΔG). The newly formed nuclei grow by aggregation of molecular species until a critical size *(*$r_{c}$), stable upon dissolution, is achieved. The nucleation rate is defined by the Arrhenius relationship as:

(4) $${B=K}_{1}\exp\left(-\frac{\Delta G_{\mathrm{cr}}}{\mathrm{KT}}\right)\;{=K}_{1}\exp\left(-\frac{16\pi \gamma^{3}v^{2}}{{3k}^{3}T^{3}{{[\ln(S}_{r})]}^{2}}\right)$$

Here, $K_{1}$ is a constant, K is Boltzmann’s constant, T is the absolute temperature, $S_{r}$ is the local supersaturation at particle surface, γ is the surface tension, v is the molar volume, and $\Delta G_{\mathrm{cr}}$ is the critical free energy for nucleation [73]. Hence, the local supersaturation (S) controls the nucleation kinetics that in the case of solvent-exchange precipitation or rapid mixing is defined as [73,75]:

(5) $$S\;\equiv \;\frac{C}{C^{*}}$$

where C is the real-time concentration and $C^{*}$ represents the saturation solubility [75]. In the LiPoNs process, the chitosan (CH) solution injected into the microfluidic platform (Figure 2a) is selected to locate the working point of the ternary system Water/AcOH/CH (99/1% *v*/*v*/0.01% *w*/*v*) at their solubility limit region [76] (Figure 2a,b, region I), so that in the microfluidic junction, when the concentration of the AcOH decreases below the saturation limit, inducing a further shift in the working point in the immiscibility gap, the supersaturation of chitosan solution occurs (Figure 2a,b, region II). Then, the chitosan concentration ${(C}_{\mathrm{CH}})$ continues to rise up to the saturation concentration ${(C}_{\mathrm{CH}}>C_{\mathrm{CH}}^{*}$) and reaches the critical nuclei concentration ($C_{\mathrm{CH}}^{n}$), where the nanoprecipitation process is triggered and almost monodisperse chitosan nuclei are formed [24,75] (Figure 2a,b, region III).

Regarding the phospholipids, they are amphiphilic molecules made up of a hydrophilic head group and double long hydrophobic tails, which make them poorly soluble in water unless they self-assemble into bilayers [77]. The formation of a vesicle can be described as a two-step self-assembly process: the aggregation of amphiphiles in bilayer fragments and then its closure into a vesicle [78]. 

For the aggregation of lipids, Marsh reported that the standard free energy of the lipid monomer transformation from water into a micelle of size m is given by [79]:

(6) $$\Delta G_{\mathrm{tr},m}^{0}{=\mu }_{\mathrm{mic},m}^{0}{-\mu }_{w}^{0}{=\mathrm{RTInX}}_{w}-\frac{\mathrm{RT}}{m}\mathrm{In}\left(\frac{X_{m}}{m}\right)$$

where $X_{w}$ and $X_{m}$ are the mole fractions of a lipid (with respect to water) in the monomer and micellar states, respectively; R is the ideal gas constant; and T is the absolute temperature. The simplest assumption is to consider the micelles monodisperse with a unique size m equal to the aggregation number of lipids ($n_{\mathrm{agg}}$), so $m=n_{\mathrm{agg}}$. For large micelles or extended bilayers (i.e., ${\;n}_{\mathrm{agg}}$→ ∞), the standard free energy of the transfer can be related to the critical micelle concentration, CMC, by:

(7) $$\Delta G_{\mathrm{tr}}^{0}{=\mathrm{RT}\;\mathrm{InX}}_{\mathrm{CMC}}$$

where CMC (in mole fraction units) is the critical micelle concentration of the lipid monomer in mole fraction units with respect to water [74,79].$\;\Delta G_{\mathrm{tr},m}^{0}$ is a negative free energy term (CMC < 1), underlying that micelle formation is a spontaneous process. The CMC is the concentration above which a further addition of solute molecules results in the formation of more aggregates while leaving the monomer concentration unchanged [80]. In LiPoNs’ microfluidic process, to guarantee the lipid micellisation process, the ternary system etOH/Water/Lipids (65/35% *v*/*v*/0.0072% *w*/*v*) is rationally selected to suddenly reduce the lipid’s solubility and reach the CMC (Figure 2a,b, region I)). Thus, simultaneously to the nanoprecipitation, the lipid molecules are no more soluble (Figure 2a,b, region II) and start to form an amphiphilic aggregate structure in the transition region close to the two-phase interface (($C_{\mathrm{Lipids}\;}{\text{>}\;C}_{\mathrm{Lipids}}^{\mathrm{CMC}}$) Figure 2a,b, region III). It is possible to predict this finite shape of amphiphiles’ self-assembly by determining the molecular packing parameter associated with their structural features. The molecular packing parameter is defined as:

(8) $$\mathrm{CPP}=\frac{v_{0}}{{\mathrm{al}}_{0}}$$

where $v_{0}$ and $l_{0}$ are the volume and length of the amphiphile’s tail and a is the surface area of the hydrophobic core of the aggregate [81]. We can hypothesize that the changes in the thermodynamic conditions controlled by the cHFF induce the formation of flexible or planar bilayers micelles [80], considering that phosphatidylcholines, which are characterised by double-chained lipids with large head-group areas and fluid chains, have a molecular packing parameter in the range ½ ≤ CPP ≤ 1. Then, these small aggregates’ spontaneous growth through coalescence in disk-like bilayer structures according to the kinetic model of a micelle–vesicle transition [78,82,83] (Figure 2a,b, region III). Generally, as the bilayer structures grow, they tend to minimize the overall line energy initially by curving themself into a spherical cup and then, to reduce the line energy further, by closing themself into a spherical vesicle [84]. However, in our dynamic microfluidic process, the further increase in the polarity of the surrounding microfluidic environment causes thermodynamic instabilities at the edges of bilayer fragments, which close upon themselves, entrapping the chitosan nanoprecipitate to eliminate the exposure of the lipid hydrocarbon tails [38] (minimising the edge energy) (Figure 2a,b, region IV). Moreover, limiting the further growth of formed chitosan nuclei, which theoretically would have continued until the CH fell to the saturation solubility $C_{\mathrm{CH}}^{*}$, thus producing bigger NPs, we stabilize the lipid bilayer coverage. From an energetic point of view, there is an increase in the system’s total energy due to the bending of the bilayers that finally disappear when vesicles are formed. Ultimately, the described process has led to the generation of LiPoNs with a stable chitosan core entrapped in a liposome structure (Figure 2a,b, Region V).

## 5. Conclusions

A one-step microfluidic process, defined as coupled Hydrodynamic Flow Focusing (cHFF), is proposed to thermodynamically guide the production of complex hybrid nanostructures. The obtained Lipid–Polymer Nanoparticles (LiPoNs), made up of a chitosan core covered by a lipid bilayer, did not only take advantage of the inherent properties of each bulk material, but their combination provides new functionality to improve nano–bio interactions.

By controlling the material conformation, the component concentrations, the solvent–antisolvent coupling and their mixing times in a microfluidic device, we modulated the compactness of the chitosan core and the extent of lipid counterpart; therefore, we present different strategies to tune the elasticity of these hybrid NPs.

LiPoNs nanoparticles, obtained at optimal conditions, with an average size of about 77 nm and a slightly negative surface charge, show excellent physical stability in different environmental conditions due to the architectural advantage of polymer core loading. Moreover, the chitosan core of LiPoNs, acting as a reservoir, enabled the high co-loading of the Gd-DTPA and Atto 633 for multimodal imaging applications.

For theranostic purposes, the cHFF strategy was exploited for the simultaneous encapsulation of Irinotecan and Gd-DTPA in LiPoNs.

The theranostic effect caused by Irinotecan and Gd-DTPA co-loaded LiPoNs was validated by investigating the cytotoxic effect on U87 MG cells upon 48 h of treatment, showing competition between the free IRI and the delivered one, speeding up the uptake and enhancing cytotoxicity at a reduced concentration.

The high cellular uptake and the biological response of active agents co-loaded LiPoNs can be connected to the peculiar elasticity and fluidity that characterised these hybrid systems. We proved that through the microfluidic approach cHFF, it is possible to achieve further control of the selected composition, triggering cell internalisation pathways, and subsequently to enhanced NPs–tissue interactions.

## Figures and Tables

**Table 1:** Table summary of average size, polydispersity index (PDI), zeta potential and co-encapsulated efficacy for different LiPoNs formulations.

	FR^2^	Average Size (nm)	PDI	Zeta Potential (mV)	EE Gd-DTPA (%)	Co-EE Active Agent (%)
LiPoNs	0.073	77.4	0.22	−17.4	/	/
Gd-DTPA LiPoNs		95.3	0.30	−11	78%	/
Atto633 LiPoNs		/	/	−3.7	/	57%
Atto633-Gd-DTPA LiPoNs		/	/	−10.9	67%	55%
IRI-Gd-DTPA LiPoNs		112.8	0.28	−14.2	79%	64%

## Data Availability

The data presented in this study are available in the Supplementary Materials.

## Supplementary Materials

The following supporting information can be downloaded at: https://www.mdpi.com/article/10.3390/biomedicines10020438/s1, Table S1: Range of process parameters performed in the preliminary experimental campaign; Figure S1: Effect of lipid concentration and etOH/Water ratio on liposomes morphology evaluated by TEM. Hydrodynamic Flow Focusing performed at FR^2^ equal to 0.51 by injecting in the side channels different Lipid concentrations for a fixed mass ratio (8:1-SPC:Chol): (a) 0.016% *w*/*v* of Lipids dissolved in etOH/Water (64/36% *v*/*v*); (b) 0.0072% *w*/*v* of Lipids dissolved in: etOH/Water (65/35% *v*/*v*) and (c) 0.0072% w/v of Lipids dissolved in etOH/Water (80/20% *v*/*v*); (d) size distribution of NPs obtained for 0.0072% *w*/*v* of Lipids dissolved in etOH/Water (80% *v*/*v*–0% *v*/*v*) and etOH/Water (65% *v*/*v*–35% *v*/*v*); Figure S2: Effect of AcOH volume percentage on liposomes’ morphology evaluated by TEM. Hydrodynamic Flow Focusing performed by injecting in the side channels Lipids (0.0072% *w*/*v*) dissolved in etOH/Water (65/35% *v*/*v*) with different percentages of AcOH in the middle channel: (a) 1% *v*/*v* and (b) 10% *v*/*v*; (c) the effect of Acetic Acid (AcOH) (1% *v*/*v*–10%*v*/*v*) on NPs’ size distribution; Figure S3: Effect of chitosan concentration on Lipid–Polymer NPs’ morphology. coupled Hydrodynamic Flow Focusing performed injecting 0.0072% *w*/*v* of Lipids into the side channels dissolved in etOH/Water (65/35% *v*/*v*), while in the middle channel, different concentrations of chitosan are dissolved in a mixture of AcOH/Water (1/99% *v*/*v*): (a) 0% *w*/*v*, (b) 0.01% *w*/*v* and (c) 0.0375% *w*/*v*; (d) the effect of chitosan concentration (0.01% *w*/*v*–0.0375%*w*/*v*) dissolved in a mixture of AcoH/Water (1/99% *v*/*v*) on NPs’ size distribution; Figure S4: Effect of FR^2^ on Lipid–Polymer NPs’ morphology. Lipids (0.0072% *w*/*v*) are injected into the side channels dissolved in etOH/Water (65/35% *v*/*v*), while the chitosan (0.01% *w*/*v*) in acid solution (1% *v*/*v*) is injected into the middle one. (a) Size distribution of LiPoNs for the different volumetric flow rate ratios (FR^2^) tested. Figure S5: Morphological characterisation of feasibility by TEM. In coupled Hydrodynamic Flow Focusing, Lipids (0.0072% *w*/*v*) are injected into the side channels dissolved in etOH/Water (65/35% *v*/*v*), while the chitosan (0.01% *w*/*v*) in acid solution (1% *v*/*v*) is injected into the middle one. TEM images stained with osmium smoke of different volumetric flow rate ratios (FR^2^) tested: (a) FR^2^ of 0.024, (b) FR^2^ of 0.073 (c), FR^2^ of 0.12 and (d) FR^2^ of 0.17. (e) Study of FR^2^ effect on nanoparticle average size, standard deviation and zeta potential [85]; Figure S6: Effect of collection volume on LiPoNs morphology. (A) Nanoparticle Size distribution of LiPoNs produced by injecting Lipids in the side channels dissolved in etOH/Water (65/35% *v*/*v*), while the chitosan (0.01% *w*/*v*) in acid solution (1% *v*/*v*) is injected into the middle one Lipids (0.0072% *w*/*v*) at FR^2^ of 0.073 for different volumes of water collection: 2 mL, 3.5 mL and 8 mL. Morphological characterisation by TEM of LiPoNs stained with osmium smoke at different water collection volumes: (B) 3.5 mL and (C) 8 mL of water [86,87,88,89]; Figure S7: (A) Cryo-TEM image and (B) confocal image of LiPoNs stained with CellMask™ Orange Plasma membrane stain (dilution 1:10^4^) performed at optimal conditions FR^2^ of 0.073 [39,40,41]; Figure S8: Structure investigation of LiPoNs: (a) Frame of NTA video of LiPoNs performed at optimal conditions diluted 1:200 in PBS; (b) Different colours represent measures of LiPoNs size distribution in function of the mean nanoparticle concentration from the five independent experiments; (c) Quantitative evaluation of LiPoNs size distribution; (d) Different colours and sizes of markers represent measures of particle size and scattered light intensity of single particle from the five independent experiments; Figure S9: Calibration curves of Gd-DTPA, Atto 633, Irinotecan. (a) Gd-DTPA calibration curve for ir sequence (1/T1) and LiPoNs, Gd-DTPA-loaded LiPoNs, Atto633-loaded LiPoNs, Atto633-Gd-DTPA co-loaded LiPoNs, IRI-Gd-DTPA co-loaded LiPoNs localisation within the curve. (b) Atto 633 calibration curve and LiPoNs, Gd-DTPA LiPoNs, Atto 633 LiPoNs, Atto633-Gd-DTPA LiPoNs and their localisation within the curve. The Atto633 calibration curve reported in the Figure S9b is a part of the complete calibration curve 25 ng/mL–4 μg/mL to graphically localize the NPs within the curve. (c) Irinotecan calibration curve and LiPoNs, Gd-DTPA LiPoNs and IRI-Gd-DTPA LiPoNs localisation within the curve. The Irinotecan calibration curve is reported in the Figure S9c is a part of the complete calibration curve 0.5–100 μM to graphically localize the NPs within the curve. Figure S10: (a) Optical imaging of Atto633-Gd-DTPA co-loaded LiPoNs by confocal microscopy; (b) Optical imaging of Atto633-Gd-DTPA LiPoNs stained with CellMask™ Orange Plasma membrane stain (dilution 1:10^4^) by confocal microscopy; (c) Merge fluorescent image of Atto633 (green) and CellMask (red) of Atto633- Gd-DTPA-loaded LiPoNs stained with CellMask™ Orange Plasma membrane stain (dilution 1:10^4^); Figure S11: Quantitative uptake of multimodal imaging LiPoNs by U87 MG cells. (a) Fluorescent Intensity (FI), (b) Forward Scattering Area (FSC) and (c) Side Scattering Area (SSC) of U87 MG cells exposed to an increasing concentration of Atto633-Gd-DTPA co-loaded LiPoNs (Lpids conc.: 30, 60 and 90 μg/mL, Atto 633 conc.: 0.2, 0.4 and 0.6 μg/mL, Gd-DTPA conc.: 75, 150 and 225 μM) for different time points: 4 h, 8 h and 24 h; Figure S12: Confocal image of U87 MG cells exposed to Atto633-Gd-DTPA co-loaded LiPoNs (Lipids conc.: 90 μg/mL, Atto 633 conc.: 0.6 μg/mL, Gd-DTPA conc.:225 μM) for 24 h in (a) transmission and (b) fluorescence (Atto 633). Figure S13. Raw flow cytometry data. Identification of PE_CY5_A fluorescence of the U87 MG cell population: (a) unexposed and (b–f) exposed to Atto633-Gd-DTPA LiPoNs (Lipids conc.: 90 μg/mL) for different time points: (b) 2 h, (c) 4 h, (d) 6 h, (e) 8 h and (f) 24 h; Figure S14: Raw flow cytometry data. Identification of PE_CY5_A fluorescence of the U87 MG cell population: (a) unexposed and (b–d) exposed to Atto633-Gd-DTPA LiPoNs (Lipids conc.: 60 μg/mL) for different time points: (b) 4 h, (c) 8 h and (d) 24 h; Figure S15: Raw flow cytometry data. Identification of PE_CY5_A fluorescence of the U87 MG cell population: (a) unexposed and (b–d) exposed to Atto633-Gd-DTPA LiPoNs (Lipids conc.: 30 μg/mL) for different time points: (b) 4 h, (c) 8 h and (d) 24 h; Video S1: NTA video of LiPoNs performed with coupled Hydrodynamic Flow Focusing at standard flow conditions: 0.0072% *w*/*v* of Lipids (molar ratio 16:4 SPC:Chol) dissolved in etOH/Water (65/35% *v*/*v*) in the side channel, while 0.01% *w*/*v* of CH dissolved in the middle channel in acetic solution (AcOH 1% *v*/*v*) and FR^2^ of 0.073; diluted 1:200 in PBS; Video S2: Merge Z-stack video of the bright-field and fluorescence (Atto 633) of U87 MG cells exposed to Atto633-Gd-DTPA-loaded LiPoNs (Lipids: 90 μg/mL, Gd-DTPA: 225 μM, Atto 633: 0.6 μg/mL) for 24 h with (a) and without (b) CellMask™ Orange Plasma membrane stain; Video S3: Merge Z-stack video of the bright-field and fluorescence (Atto 633)) of U87 MG cells exposed to Atto633-Gd-DTPA-loaded LiPoNs (Lipids: 90 μg/mL, Gd-DTPA: 225 μM, Atto 633: 0.6 μg/mL) for 24 h.
