# Supplementary material for: coupled Hydrodynamic Flow Focusing (cHFF) to Engineer Lipid–Polymer Nanoparticles (LiPoNs) for Multimodal Imaging and Theranostic Applications
Source: Biomedicines. 2022 Feb 14;10(2):438. doi: 10.3390/biomedicines10020438 (PMC8962394; doi:10.3390/biomedicines10020438)
Supplement: Supplementary file 1 [file biomedicines-10-00438-s001.zip › biomedicines-1577652-Supplementary Materials.pdf]

Supplementary Materials

coupled Hydrodynamic Flow Focusing (cHFF) to Engineer Lipid-Polymer Nanoparticles (LiPoNs) for Multimodal Imaging and Theranostic applications

Felicia Roffo [1,2], Alfonso Maria Alfonso Maria Ponsiglione[1], Paolo Antonio Netti[1,2,3] and Enza Torino [1,2,3,*]

[1] University of Naples Federico II, Department of Chemical, Materials and Production Engineering (DICMaPI), P.le Tecchio 80, 80125, Naples, Italy; felicia.roffo@unina.it (F.R.); alfonsomaria.ponsiglione@unina.it (A.M.P.); nettipa@unina.it (P.A.N.)
[2] Interdisciplinary Research Center on Biomaterials, CRIB, University of Naples Federico II, P.le Tecchio 80, 80125, Naples, Italy
[3] Center for Advanced Biomaterials for Health Care, CABHC, Istituto Italiano di Tecnologia, IIT@CRIB, Largo Barsanti e Matteucci 53, 80125 Naples, Italy
* Correspondence: enza.torino@unina.it

1. Experimental Campaign

Table S1. Range of process parameters performed in the preliminary experimental campaign.

<table>
<tr><th>SPC:Chol Mass ratio</th><th>Lipids (% w/v)</th><th>etOH/Water ratio (% v/v)</th><th>AcOH/Water ratio (% v/v)</th><th>CH (% w/v)</th><th>Collection Volume (mL)</th><th>FR²</th><th>Middle Flow: Side Flow (μL/min)</th><th>Average Size (nm)</th><th>PDI</th></tr>
<tr><td rowspan="17">8:1</td><td>0.016</td><td>64/36</td><td>/</td><td>/</td><td>4</td><td rowspan="7">0.51</td><td rowspan="7">21:41</td><td>/</td><td>/</td></tr>
<tr><td rowspan="16">0.0072</td><td>80/20</td><td>/</td><td>/</td><td rowspan="11">2</td><td>177.2</td><td>0.503</td></tr>
<tr><td rowspan="15">65/35</td><td>/</td><td>/</td><td>126.9</td><td>0.401</td></tr>
<tr><td>10/90</td><td>/</td><td>204.3</td><td>0.283</td></tr>
<tr><td rowspan="13">1/99</td><td>/</td><td>225</td><td>0.394</td></tr>
<tr><td>0.0375</td><td>242.8</td><td>0.569</td></tr>
<tr><td rowspan="11">0.01</td><td>131</td><td>0.554</td></tr>
<tr><td>0.68</td><td>28:41</td><td>186.7</td><td>0.534</td></tr>
<tr><td>0.34</td><td>14:41</td><td>199.7</td><td>0.367</td></tr>
<tr><td>0.17</td><td>7:41</td><td>134.1</td><td>0.491</td></tr>
<tr><td>0.073</td><td>3:41</td><td>66.79</td><td>0.269</td></tr>
<tr><td>0.024</td><td>1:41</td><td>100.2</td><td>0.349</td></tr>
<tr><td rowspan="4">3.5</td><td>0.17</td><td>7:41</td><td>87.14</td><td>0.150</td></tr>
<tr><td>0.12</td><td>5:41</td><td>88.22</td><td>0.148</td></tr>
<tr><td>0.073</td><td>3:41</td><td>77.45</td><td>0.218</td></tr>
<tr><td>0.024</td><td>1:41</td><td>110.5</td><td>0.157</td></tr>
<tr><td>8</td><td>0.073</td><td>3:1</td><td>308.7</td><td>0.337</td></tr>
</table>

Notes: Ethanol (etOH), Acetic Acid (AcOH), Soybean Phosphatidylcholine (SPC), Cholesterol (Chol), Lipids (mass ratio 8:1 SPC:Chol), chitosan (CH), FR^2 (Flow rate ratio).

1.1. Study of the concentration of the reagents and of solvent–nonsolvent ratio for the production of LiPoNs

A preliminary study was performed (Table S1) evaluating the concentration of the reagents (lipid concentration: 0.016 and 0.0072% *w/v* and chitosan concentration: 0.01% *w/v* and 0.0375% *w/v*) and the effect of solvent–nonsolvent ratio (EtOH/Water: 80/20% *v/v* and 65/35% *v/v* and AcOH/Water: 10/90% *v/v* and 1/99 % *v/v*) on the morphology of LiPoNs and their physiochemical properties at fixed FR^2 of 0.51 (obtained at 21 μL/min in the middle phase and 41 μL/min in the side phase) and water collection volume of 2mL.

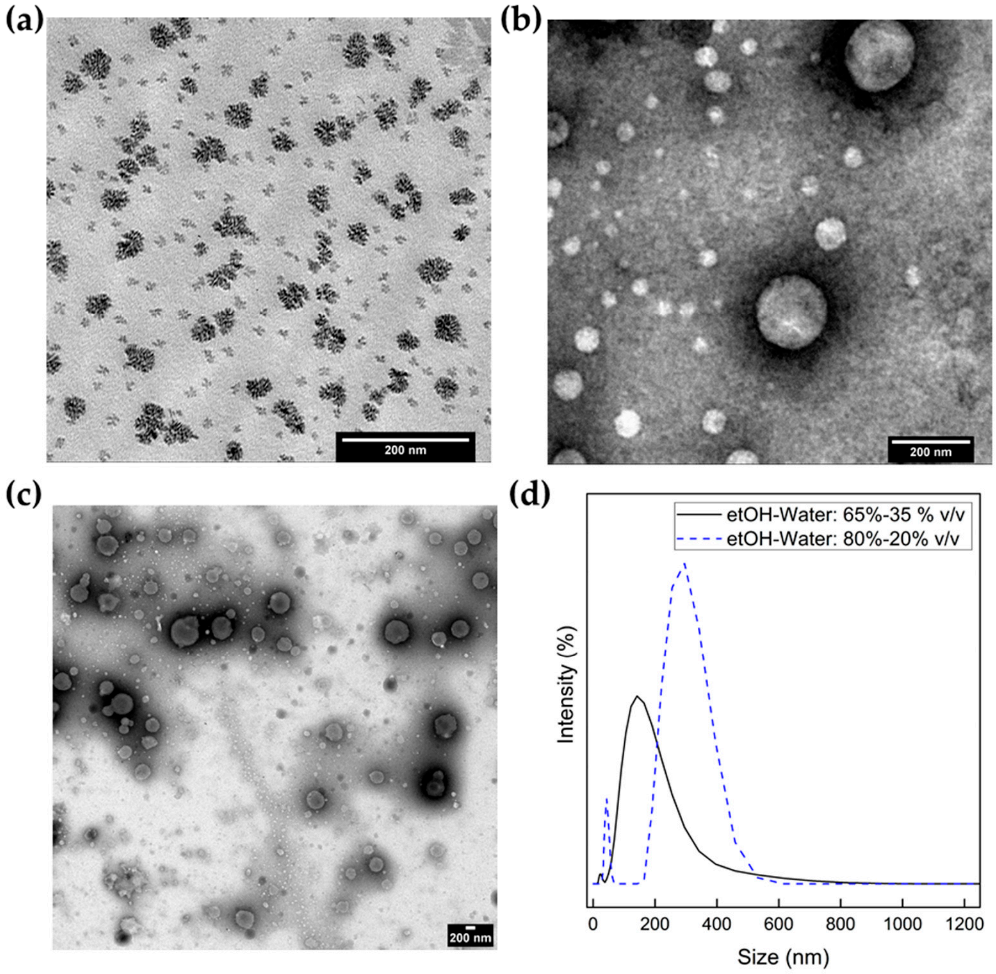

Figure S1. Effect of lipids concentration and EtOH/Water ratio on liposomes morphology evaluated by TEM. Hydrodynamic Flow Focusing at FR^2 equal to 0.51 performed by injecting in the side channels different Lipids concentration for a fixed mass ratio (8:1-SPC:Chol). (**a**) 0.016% *w/v* of Lipids dissolved in etOH/Water (64/36% *v/v*); (**b**) 0.0072% *w/v* of Lipids dissolved in etOH/Water (65/35% *v/v*) and (**c**) 0.0072% *w/v* of Lipids dissolved in etOH/Water (80/20% *v/v*); (**d**) Size distribution of NPs obtained for 0.0072% *w/v* of lipids dissolved in etOH/Water (80/20% *v/v*) and etOH/Water (65/35 % *v/v*).

Firstly, the lipids, at fixed composition (mass ratio 8:1-SPC:Chol), were dissolved in etOH/Water (65-64/35-36% *v/v*) at two different concentrations (0.016 and 0.0072% *w/v*) and were injected from the side channels sheeting the middle water stream (at FR^2 equal to 0.51). As shown in Figure S1a for lipids concentration of 0.016% *w/v* an uncontrolled precipitation was observed, while the formation of liposomes structure was obtained for a Lipids concentration of 0.0072% *w/v* (Figure 2b). To evaluate the effect of etOH/Water percentage on the production of liposomes, the Lipids at 0.0072% *w/v* were dissolved in a different etOH/Water ratio (80/20% *v/v*) and injected into the microfluidic device (Figure S1c). Smaller liposomes, as observed in Figure S1b and reported in DLS distribution (Figure S1d), were obtained for the lower ethanol fraction (etOH/Water -65/35% *v/v*) due to enhanced ethanol extraction at microfluidics focusing. Therefore, the etOH/Water mixture at 65/35% *v/v* was selected for subsequent studies.

Then, still at constant FR^2 of 0.51, the effect of the acetic acid (1% *v/v* and 10% *v/v*) and the chitosan addition at different concentration (0.01% *w/v* and 0.0375% *w/v*) on the liposome structure was evaluated in Figures S2 and S3. The absence of massive precipitation, combined with the evaluation of the morphologies obtained by

TEM images (Figures S2a,b and S3a–c) and DLS data (Figures S2c–S3d), were used to identify the reagent concentrations (0.0072% *w/v* of Lipids - 0.01% *w/v* of chitosan) and the solvent-non solvent ratios (etOH/Water 65/35% *v/v*, AcOH/Water 1/99% *v/v*) for successive studies.

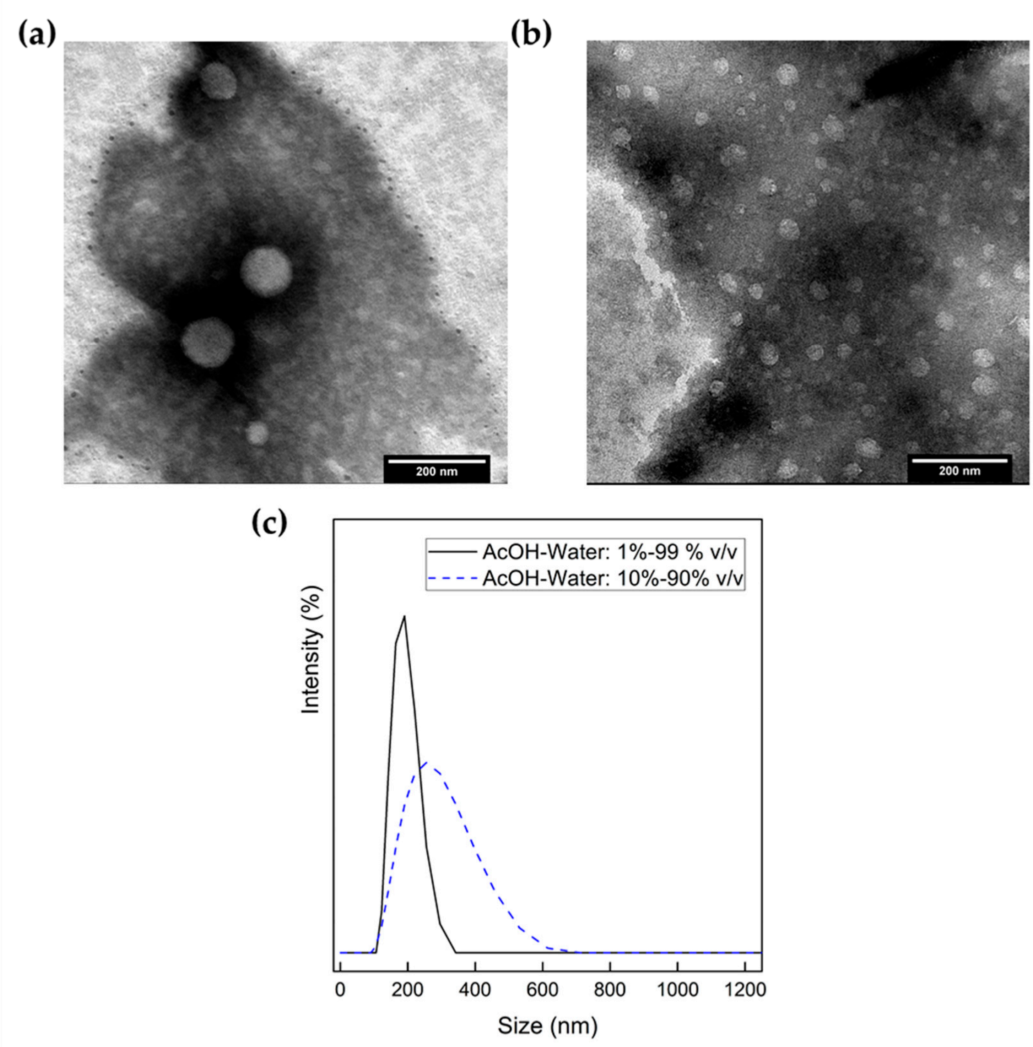

Figure S2. Effect of AcOH volume percentage on liposomes morphology evaluated by TEM. Hydrodynamic Flow Focusing performed by injecting in the side channels Lipids (0.0072% *w/v)* dissolved in etOH/Water (65/35% *v/v*) with different percentage of Acetic Acid (AcOH) in the middle channel: (a) 1% *v/v* and (b) 10% *v/v.* (c) The effect of AcOH (1% *v/v*- 10% *v/v*) on NPs' size distribution.

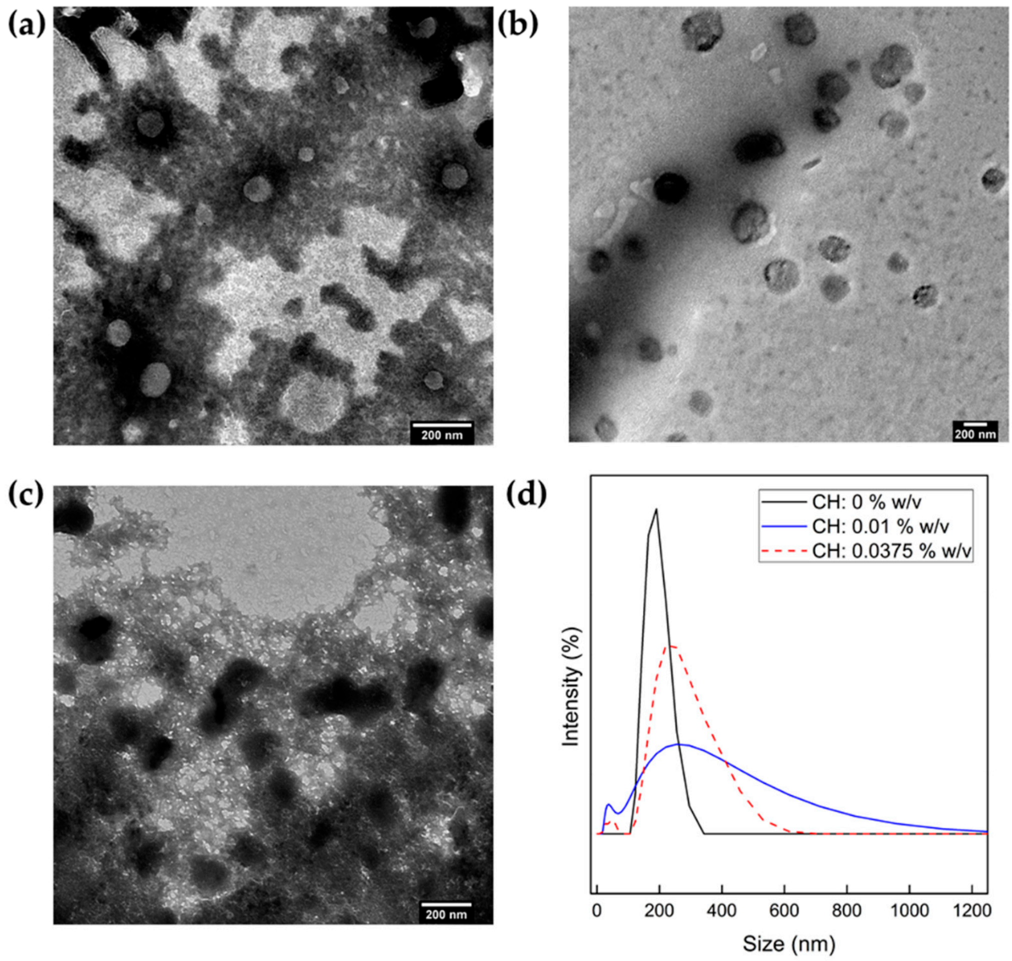

Figure S3. Effect of chitosan concentration on Lipid-Polymer NPs' morphology. coupled Hydrodynamic Flow Focusing performed injecting 0.0072% w/v of Lipids in the side channels dissolved in etOH/Water (65/35% *v/v*),while in the middle channel different concentrations of chitosan are dissolved in a mixture of AcOH/Water at 1% *v/v*): (**a**) 0% *w/v*, (**b**) 0.01% *w/v* and (**c**) 0.0375% *w/v*. (**d**) The effect of chitosan concentration (0.01% *w/v* and 0.0375% *w/v*) dissolved in a mixture of AcoH/Water (1/99% *v/v*) on NPs' size distribution.

1.2. The effect of FR² on the morphology of LiPoNs

The effect of FR² is studied by keeping constant the reagent concentration (0.0072% *w/v* of Lipids and 0.01% *w/v* of chitosan) and the solvent-non solvent ratios (etOH/Water: 65/35% *v/v* and AcOH/Water: 1/99% *v/v),* optimised in previous paragraphs. The role FR² on the morphology of LiPoNs nanostructure were observed by varying the FR², obtained at constant side flow rate of 41 µL/min and changing the middle flowrate alternatively (1-3-7-14-21-28 µL/min), always keeping constant the collection volume equal to 2 mL of water. The DLS distributions in Figure S4a show a monodisperse population of nanoparticle for lower FR² of 0.024 and 0.073, while a bimodal distribution is reported for higher FR² from 0.17 to 0.68.

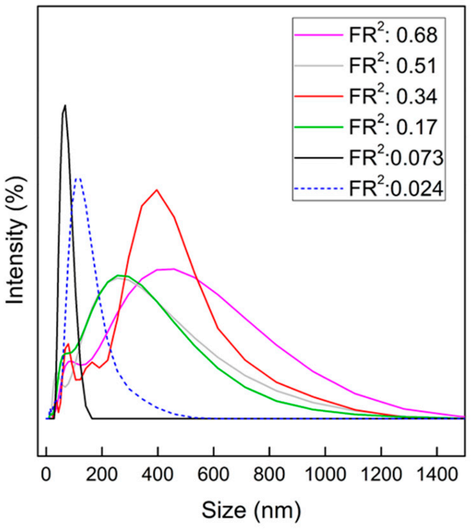

Figure S4. Effect of FR^2 on Lipid-Polymer NPs morphology. Lipids (0.0072% *w/v*) are injected into the side channels dissolved in etOH/Water (65/35% *v/v*), while the chitosan (0.01% w/v) in acid solution (1% *v/v*) is injected into the middle one. (**a**) Size distribution of LiPoNs for different volumetric flow rate ratios (FR^2).

The previous experimental campaign allows the definition of a reduced operative window for the FR^2 ranging from 0.024 to 0.17. A further study was conducted in this window to analyse the role of solvent displacement and residence time distribution on the morphology of nanoparticles. A more sensitive variation of FR^2 is, therefore, performed to enlarge the phenomena in the range from 0.024 to 0.17, obtained by setting middle channel flow rates from 1 μL/min to 7 μL/min (with constant stepwise of 2 μL/min), while side channel was kept constant at 41 μL/min (Figure S5).

Decreasing FR^2 down to 0.024 leads to a reduction in the τ_{mix} of the components. The reduction in the polymer flow rate induces a size reduction of the chitosan nuclei formation, but also it slows down the ethanol extraction and, therefore, the formation of the bilayer structures. The results reported in Figure S5a,b show a massive production of nanostructures of different morphology. A clear excess of lipidic coating is identified in Figure S5a, confirming the assembly of the bilayer structures, while a more homogenous coating is observed in Figure S5b. For FR^2 at 0.073, obtained at middle channel flow rate of 3 μL/min and side channel flow rate at 41 μL/min, the extraction time is adequate for lipid fragments to aggregate [1] and then cover the polymer structures.

By increasing the FR^2 up to 0.17, obtained by changing the middle flow rate, we observed the formation of chitosan precipitates as uncontrolled morphologies and polymer-coated structures (Figure S5c,d). Indeed, in Figure S5d, it is possible to distinguish bilayer fragments in a bending or straight lipid structure formed separately from precipitated chitosan, confirming an un-coupled mechanism. Moreover, the reduction in chitosan entrapment within the LiPoNs complex is also confirmed by the positive zeta potential (44.2 mV) for the higher flow rate ratio (0.17), reported in Figure S5e.

Consequently, the value of FR^2 of 0.073 (obtained at a middle flow rate of 3 μL/min, a side flow rate of 41 μL/min), a chitosan concentration of 0.01% *w/v* (dissolved in acetic solution 1% *v/v*- middle phase) and a Lipid concentration of 0.0072% *w/v* (dissolved in etOH/Water- 65/35% *v/v*- side phase) were selected at *optimal conditions* and were further used for the next experiments.

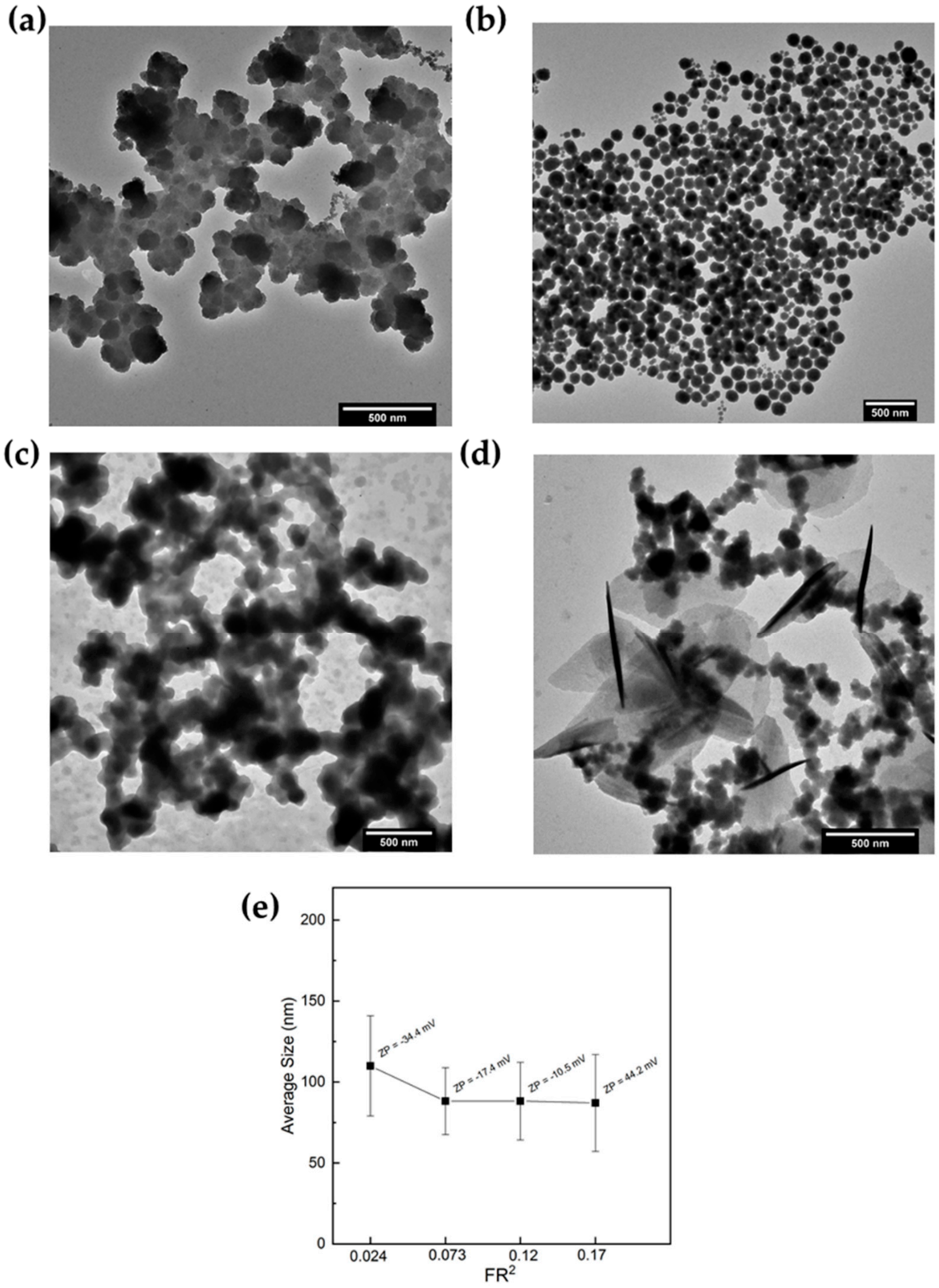

Figure S5. Morphological characterisation of feasibility study by TEM. In coupled Hydrodynamic Flow Focusing, Lipids (0.0072% *w/v*) are injected into the side channels dissolved in etOH/Water (65/35% *v/v*), while the chitosan (0.01% *w/v*) in acid solution (1% *v/v*) is injected into the middle one. TEM images stained with osmium smoke of different volumetric flow rate ratios (FR^2) tested: (**a**) FR^2 of 0.024, (**b**) FR^2 of 0.073 **(c),** FR^2 of 0.12 and (**d**) FR^2 of 0.17. (**e**) Study of FR^2 effect on the nanoparticle's average size and zeta potential.

1.3. The effect of the collection volume on the morphology of LiPoNs

In the following steps, we investigated the influence of the collection volume on liposome morphology at fixed FR^2 of 0.073 and *optimal conditions*. DLS measurement and TEM images show an enlargement of LiPoNs' size as the collection volume increases up to 8 mL (2, 3.5 and 8 mL) (Figure S6a). In this step, the collection volume was firstly set at 3.5 mL instead of 2 mL to reduce the residual ethanol in the final formulation and prevent re-solubilisation of formed lipid–polymer structures since no effect on the final LiPoN size was observed due to

this variation (Figure S6a). However, an increase in the collection volume to 8mL produces an increase in size. Indeed, the rapid change in pH around the liposome, as they are produced, could lead to a diffusion of water inside the liposome to balance the pH difference created between the newly formed chitosan core and neutral pH of the collection volume [2–4]. Moreover, at the lower collection volume of 3.5 mL, the high residual ethanol amount could decrease the water permeability by replacing the water in the hydration shells of the head groups and accumulating itself in transient defects in the hydrophobic part of the bilayer [5]. The assumption perfectly matches the irregular and swelled shape of the LiPoNs observed in the TEM images (Figure S6b–c) obtained at 3.5 and 8 mL.

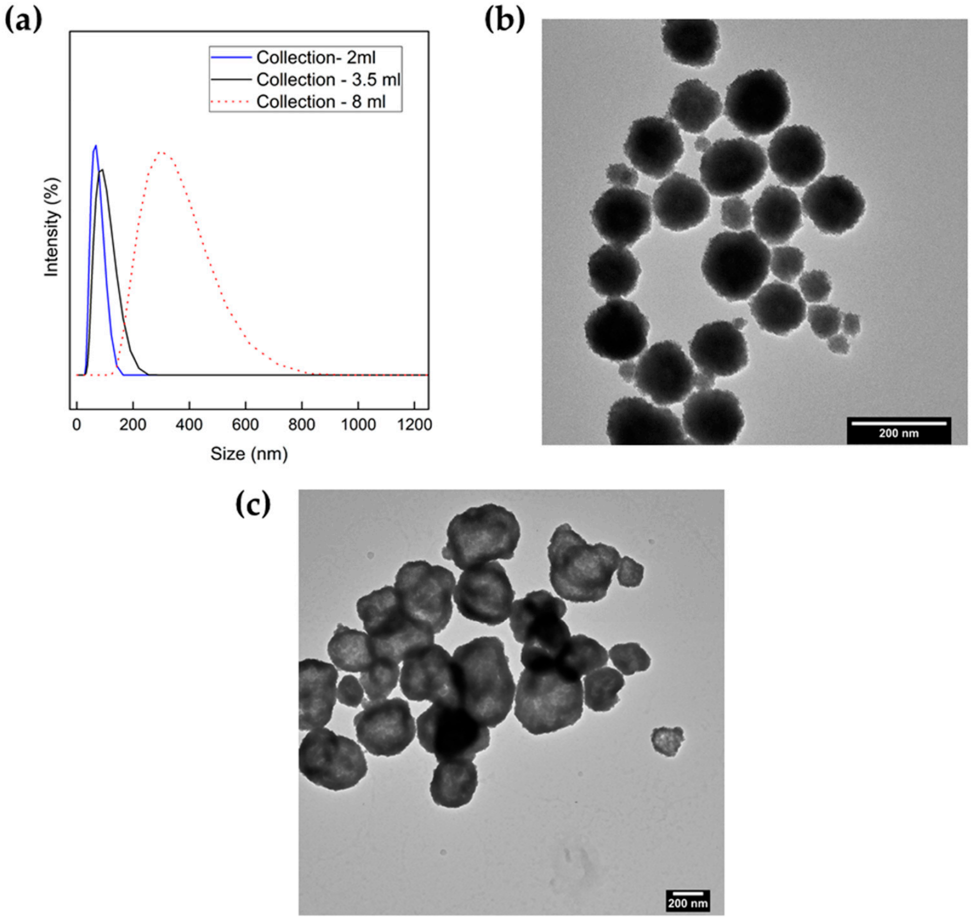

Figure S6. Effect of collection volume on LiPoNs morphology. **(a)** Nanoparticle size distribution of LiPoNs produced by injecting Lipids in the side channels dissolved in etOH/Water (65%35% *v/v*), while the chitosan (0.01% *w/v*) in acid solution (1% *v/v*) is injected into the middle one Lipids (0.0072% *w/v*) at FR^2 of 0.073 for different volumes of water collection: 2 mL, 3.5 mL and 8 mL. Morphological characterisation by TEM of LiPoNs stained with osmium smoke at different water collection volumes: (**b**) 3.5 mL and (**c**) 8 mL of water.

These results highlight the importance, as reported in recent studies [6–8], of considering the effect of the whole microfluidic channel path on NPs' morphologies and not only at the microfluidic junction. Indeed, the length of the microfluidic chip should be suitable for completing the mixing among solvents to avoid incomplete or disassemble processes in nanoparticle formation.

Further results obtained at optimal conditions for a collection volume of 3.5 mL are reported in Figures S7–S15.

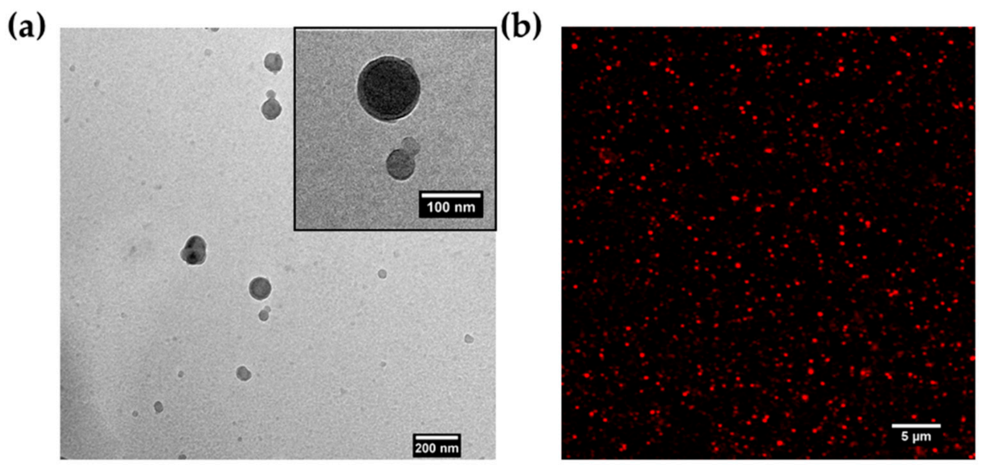

Figure S7. (**a**) Cryo-TEM image and (**b**) confocal image of LiPoNs stained with CellMask™ Orange Plasma membrane stain (dilution 1:10^4) performed at *optimal conditions* FR^2 of 0.073.

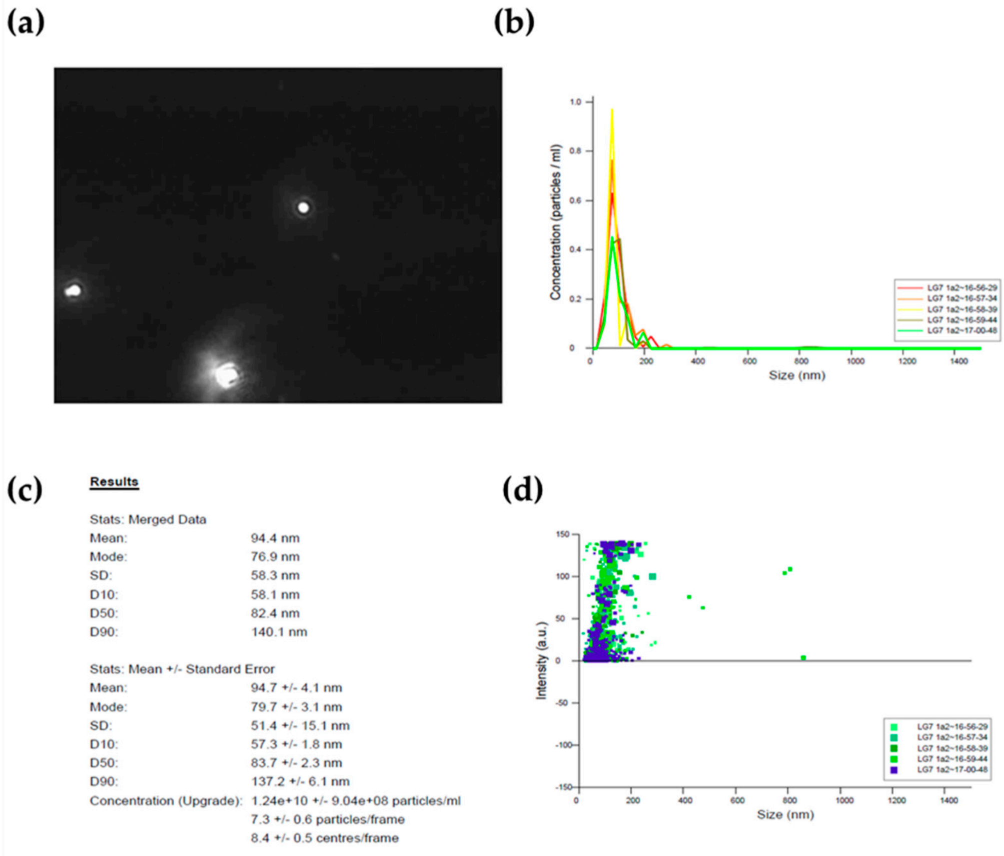

Figure S8. Structure investigation of LiPoNs: (**a**) Frame of NTA video of LiPoNs performed at *optimal conditions* diluted 1:200 in PBS; (**b**) Different colours represent measures of LiPoNs' size distribution in function of the mean nanoparticle concentration from five independent experiments; (**c**) Quantitative evaluation of LiPoNs' size distribution; (**d**) Different colours and sizes of markers represent measures of particle size and scattered light intensity of single particle from the five independent experiments.

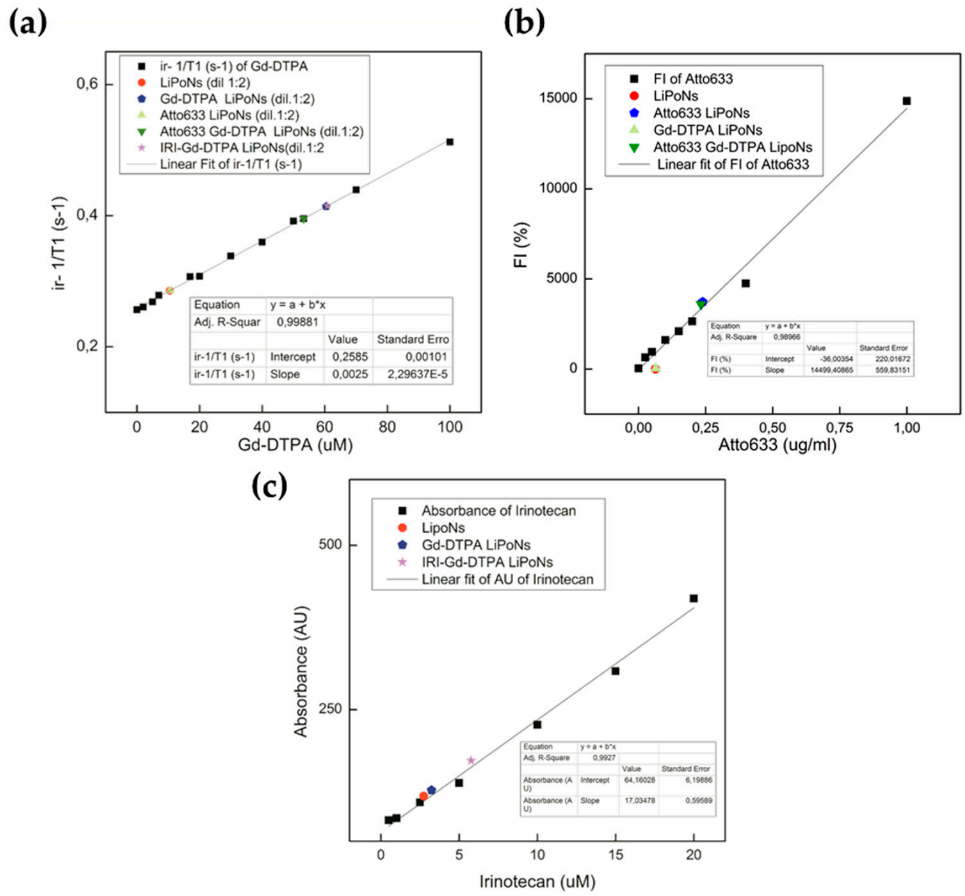

Figure S9. Calibration curves of Gd-DTPA, Atto 633, Irinotecan: (**a**) Gd-DTPA calibration curve (dispersed in water) for ir sequence (1/T1) and LiPoNs, Gd-DTPA-loaded LiPoNs, Atto633-loaded LiPoNs, Atto633-Gd-DTPA co-loaded LiPoNs, IRI-Gd-DTPA co-loaded LiPoNs localisation within the curve. (**b**) Atto 633 calibration curve and LiPoNs, Gd-DTPA LiPoNs, Atto 633 LiPoNs, Atto633-Gd-DTPA LiPoNs and their localisation within the curve. The Atto633 calibration curve reported in the figure S9b is a part of the complete calibration curve 25 ng/mL–4 μg/mL to graphically localize the NPs within the curve. (**c**) Irinotecan calibration curve and LiPoNs, Gd-DTPA LiPoNs and IRI-Gd-DTPA LiPoNs localisation within the curve. The Irinotecan calibration curve is reported in the figure S9c is a part of the complete calibration curve 0.5–100 μM to graphically localize the NPs within the curve.

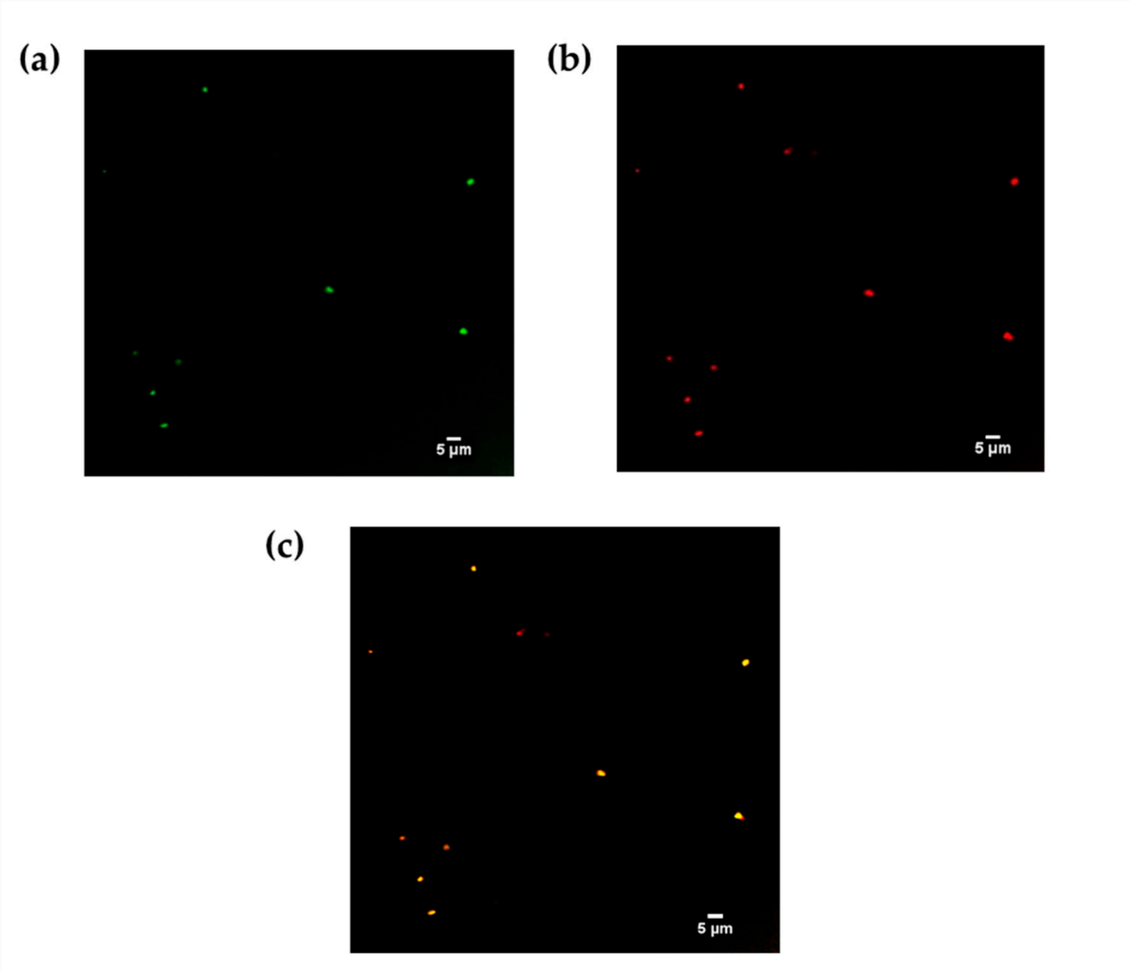

Figure S10. (**a**) Optical imaging of Atto633-Gd-DTPA co-loaded LiPoNs by confocal microscopy; (**b**) Optical imaging of Atto633-Gd-DTPA LiPoNs stained with CellMask™ Orange Plasma membrane stain (dilution 1:10^4) by confocal microscopy; (**c**) Merge fluorescent image of Atto633 (green) and CellMask (red) of Atto633- Gd-DTPA-loaded LiPoNs stained with CellMask™ Orange Plasma membrane stain (dilution 1:10^4).

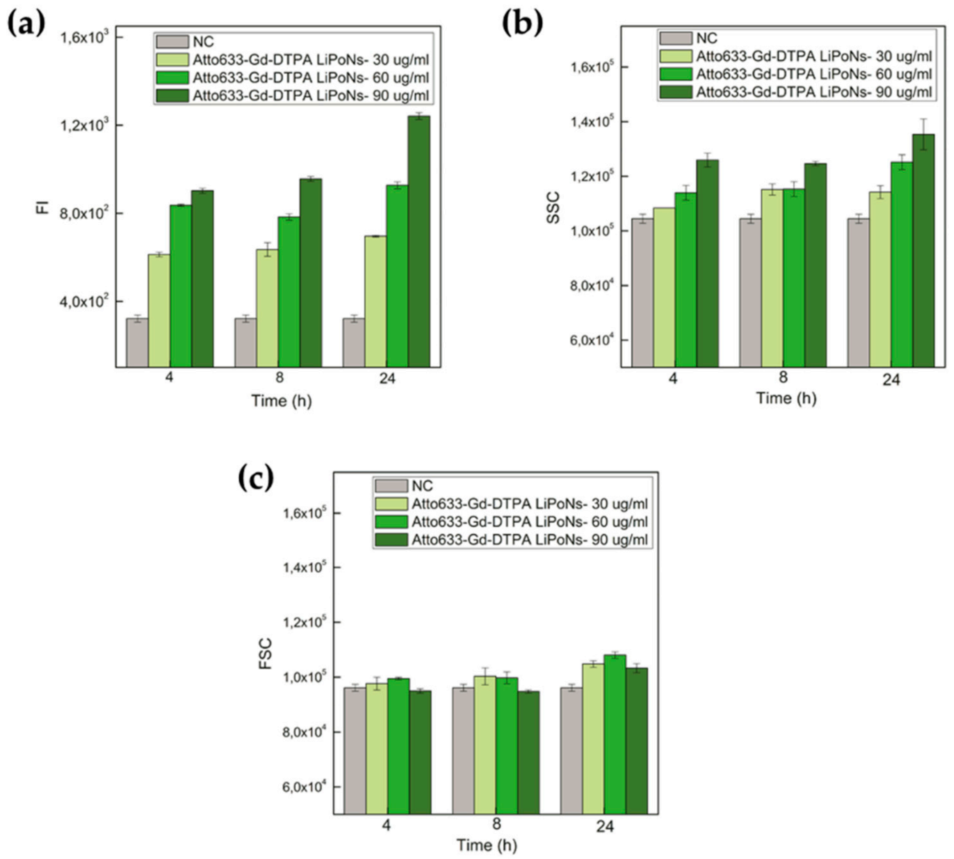

Figure S11. Quantitative uptake of multimodal imaging LiPoNs by U-87 MG cells. (**a**) Fluorescent Intensity (FI), (**b**) Forward Scattering Area (FSC) and (**c**) Side scattering Area (SSC) of U-87 MG cells exposed to an increasing concentration of Atto633-Gd-DTPA co-loaded LiPoNs Lipids (Lipid conc: 30, 60 and 90 μg/mL, Atto 633 conc.: 0.2, 0.4 and 0.6 μg/mL, Gd-DTPA conc.: 75-150-225 μM) for different time points: 4 h, 8 h, 24 h.

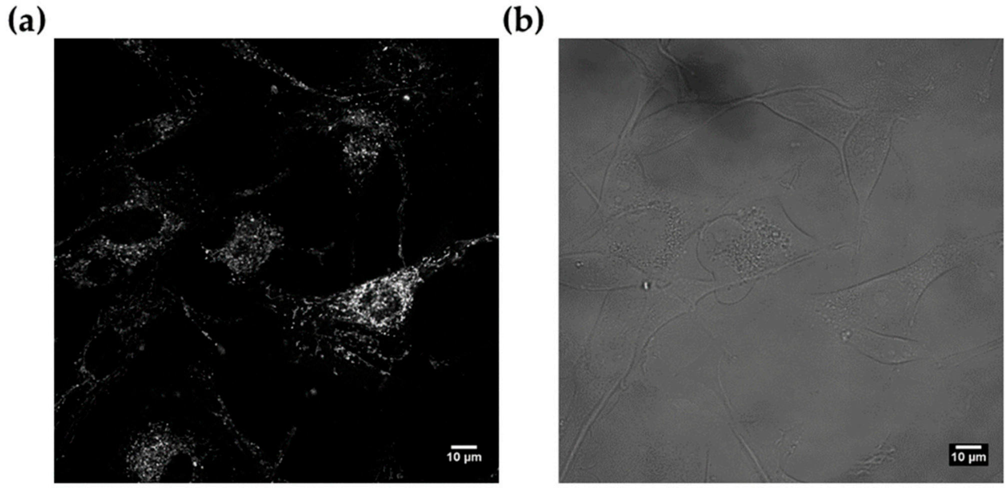

Figure S12. Confocal image of U-87 MG cells exposed to Atto633-Gd-DTPA co-loaded LiPoNs Lipids (lipid conc.: 90 μg/mL, Atto 633 conc.: 0.6 μg/mL, Gd-DTPA conc.: 225 μM) for 24 h in (**a**) transmission and (**b**) fluorescence (Atto 633).

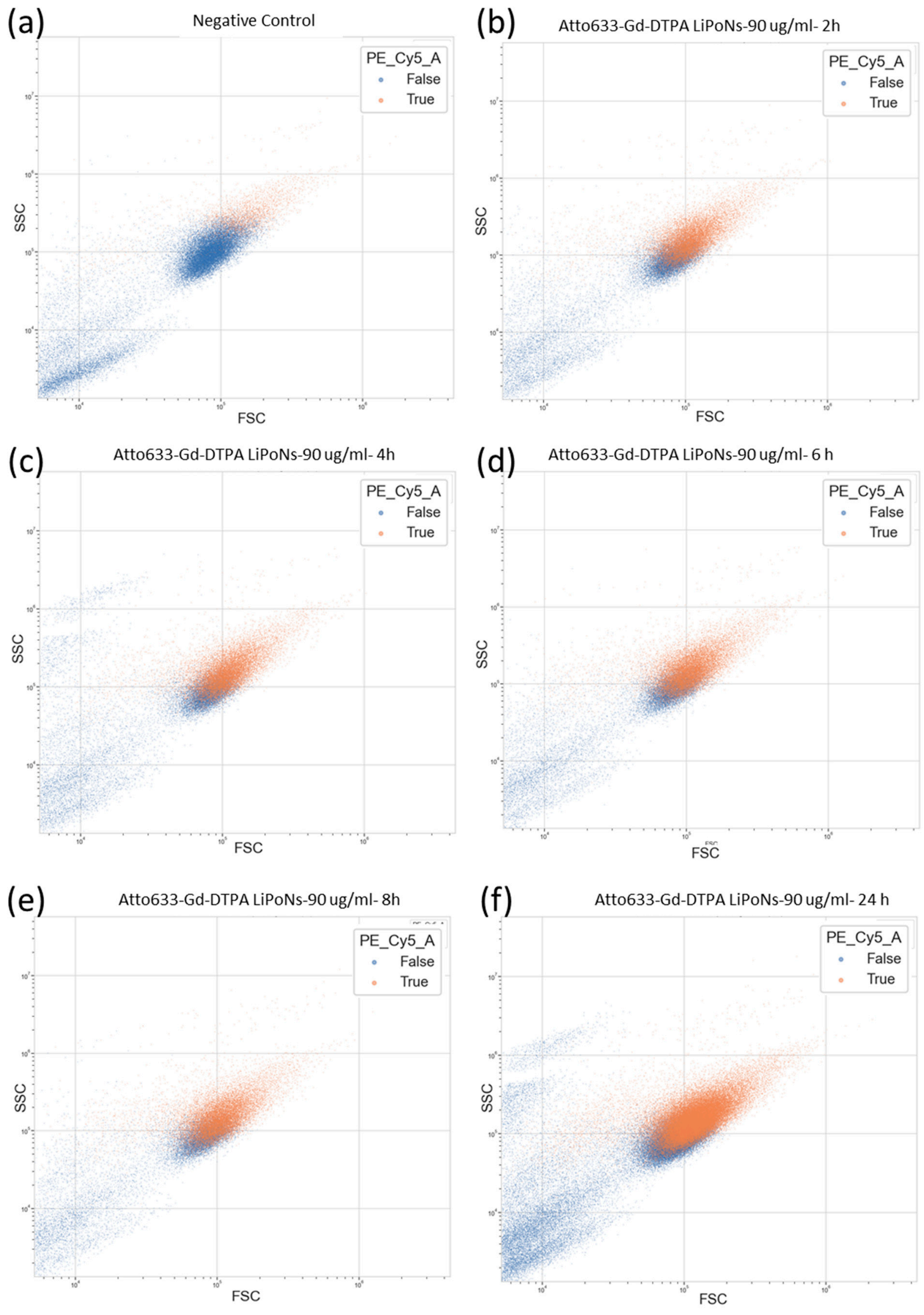
(a)
Negative Control
PE_Cy5_A
False
True
SSC
FSC
(b)
Atto633-Gd-DTPA LiPoNs-90 ug/ml- 2h
PE_Cy5_A
False
True
SSC
FSC
(c)
Atto633-Gd-DTPA LiPoNs-90 ug/ml- 4h
PE_Cy5_A
False
True
SSC
FSC
(d)
Atto633-Gd-DTPA LiPoNs-90 ug/ml- 6 h
PE_Cy5_A
False
True
SSC
FSC
(e)
Atto633-Gd-DTPA LiPoNs-90 ug/ml- 8h
PE_Cy5_A
False
True
SSC
FSC
(f)
Atto633-Gd-DTPA LiPoNs-90 ug/ml- 24 h
PE_Cy5_A
False
True
SSC
FSC

Figure S13. Raw flow cytometry data. Identification of PE_CY5_A fluorescence of the U-87 MG cell population: (**a**) unexposed and (**b–f**) exposed to Atto633-Gd-DTPA LiPoNs (Lipids conc.: 90 µg/mL) for different time points: (**b**) 2 h, (**c**) 4 h, (**d**) 6 h, (**e**) 8 h and (**f**) 24 h.

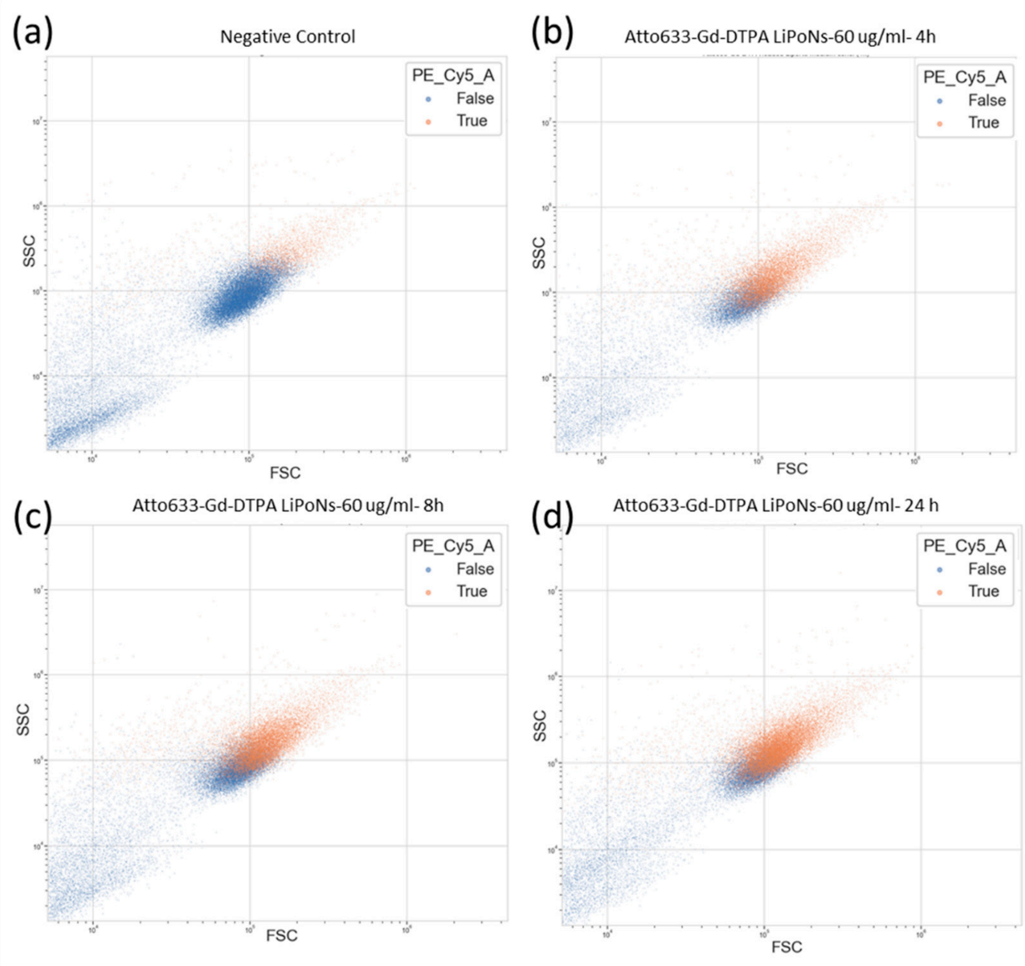

Figure S14. Raw flow cytometry data. Identification of PE_CY5_A fluorescence of the U-87 MG cell population: (**a**) unexposed and (**b–d**) exposed to Atto633-Gd-DTPA LiPoNs (Lipids conc.: 60 µg/ml) for different time points: (**b**) 4 h, (**c**) 8 h and (**d**) 24 h.

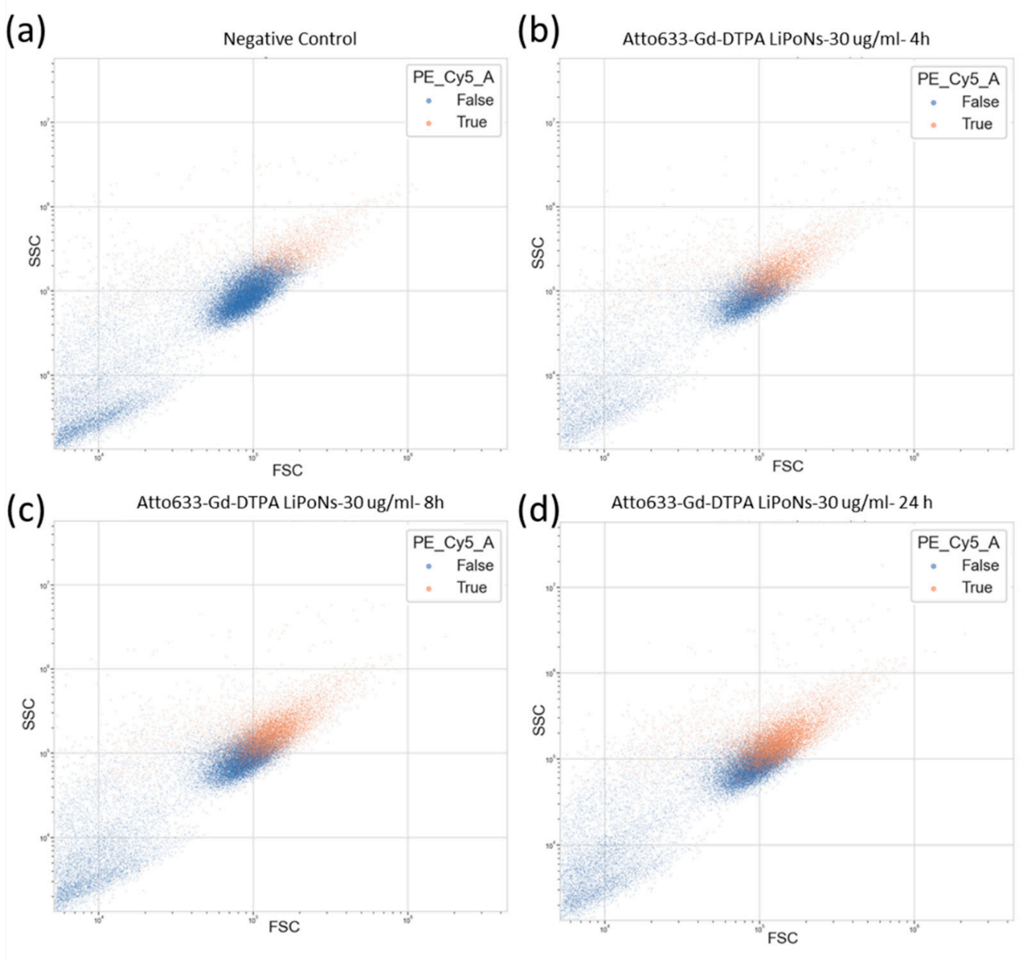

Figure S15. Raw flow cytometry data. Identification of PE_CY5_A fluorescence of the U-87 MG cell population: (**a**) unexposed and (**b–d**) exposed to Atto633-Gd-DTPA LiPoNs (Lipids conc.: 30 μg/mL) for different time points: (**b**) 4 h, (**c**) 8 h and (**d**) 24 h.

References

1. Jahn, A.; Lucas, F.; Wepf, R.A.; Dittrich, P.S. Freezing Continuous-Flow Self-Assembly in a Microfluidic Device: Toward Imaging of Liposome Formation. Langmuir 2013, 29, 1717-1723, doi:10.1021/la303675g.
2. Tazina, E.V.; Kostin, K.V.; Oborotova, N.A. SPECIFIC FEATURES OF DRUG ENCAPSULATION IN LIPOSOMES (A REVIEW). Pharmaceutical Chemistry Journal 2011, 45, 481-490, doi:10.1007/s11094-011-0661-4.
3. Gabba, M.; Frallicciardi, J.; van't Klooster, J.; Henderson, R.; Syga, L.; Mans, R.; van Maris, A.J.A.; Poolman, B. Weak Acid Permeation in Synthetic Lipid Vesicles and Across the Yeast Plasma Membrane. Biophysical Journal 2020, 118, 422-434, doi:10.1016/j.bpj.2019.11.3384.
4. Monteiro, N.; Martins, A.; Reis, R.L.; Neves, N.M. Liposomes in tissue engineering and regenerative medicine. Journal of the Royal Society Interface 2014, 11, doi:10.1098/rsif.2014.0459.
5. Jansen, M.; Blume, A. A COMPARATIVE-STUDY OF DIFFUSIVE AND OSMOTIC WATER PERMEATION ACROSS BILAYERS COMPOSED OF PHOSPHOLIPIDS WITH DIFFERENT HEAD GROUPS AND FATTY ACYL CHAINS. Biophysical Journal 1995, 68, 997-1008, doi:10.1016/s0006-3495(95)80275-4.
6. Abdelkarim, M.; Abd Ellah, N.H.; Elsabahy, M.; Abdelgawad, M.; Abouelmagd, S.A. Microchannel geometry vs flow parameters for controlling nanoprecipitation of polymeric nanoparticles. Colloids and Surfaces a-Physicochemical and Engineering Aspects 2021, 611, doi:10.1016/j.colsurfa.2020.125774.
7. Lallana, E.; Donno, R.; Magri, D.; Barker, K.; Nazir, Z.; Treacher, K.; Lawrence, M.J.; Ashford, M.; Tirelli, N. Microfluidic-assisted nanoprecipitation of (PEGylated) poly (D,L-lactic acid-co-caprolactone): Effect of macromolecular and microfluidic parameters on particle size and paclitaxel encapsulation. International Journal of Pharmaceutics 2018, 548, 530-539, doi:10.1016/j.ijpharm.2018.07.031.
8. Donno, R.; Gennari, A.; Lallana, E.; De La Rosa, J.M.R.; d'Arcy, R.; Treacher, K.; Hill, K.; Ashford, M.; Tirelli, N. Nanomanufacturing through microfluidic-assisted nanoprecipitation: Advanced analytics and structure-activity relationships. International Journal of Pharmaceutics 2017, 534, 97-107, doi:10.1016/j.ijpharm.2017.10.006.
